# A Multi-Objective Carnivorous Plant Algorithm for Solving Constrained Multi-Objective Optimization Problems

**DOI:** 10.3390/biomimetics8020136

**Published:** 2023-03-26

**Authors:** Yufei Yang, Changsheng Zhang

**Affiliations:** Software College, Northeastern University, Shenyang 110169, China

**Keywords:** carnivorous plant algorithm, constrained multi-objective optimization, cross-pollination, quasi-reflection learning, quadratic interpolation

## Abstract

Satisfying various constraints and multiple objectives simultaneously is a significant challenge in solving constrained multi-objective optimization problems. To address this issue, a new approach is proposed in this paper that combines multi-population and multi-stage methods with a Carnivorous Plant Algorithm. The algorithm employs the ϵ-constraint handling method, with the ϵ value adjusted according to different stages to meet the algorithm’s requirements. To improve the search efficiency, a cross-pollination is designed based on the trapping mechanism and pollination behavior of carnivorous plants, thus balancing the exploration and exploitation abilities and accelerating the convergence speed. Moreover, a quasi-reflection learning mechanism is introduced for the growth process of carnivorous plants, enhancing the optimization efficiency and improving its global convergence ability. Furthermore, the quadratic interpolation method is introduced for the reproduction process of carnivorous plants, which enables the algorithm to escape from local optima and enhances the optimization precision and convergence speed. The proposed algorithm’s performance is evaluated on several test suites, including DC-DTLZ, FCP, DASCMOP, ZDT, DTLZ, and RWMOPs. The experimental results indicate competitive performance of the proposed algorithm over the state-of-the-art constrained multi-objective optimization algorithms.

## 1. Introduction

Constrained multi-objective optimization problems (CMOPs) [1,2] are prevalent in many real-world applications, such as software-defined networks [3], path planning [4], wireless sensors deployment [5], and real-time systems [6]. Generally, a minimized CMOP can be defined as Equation (1).

(1) $$\min_{x\in S}f\left(x\right)={\left(f_{1}\left(x\right),f_{2}\left(x\right),\ldots ,f_{m}\left(x\right)\right)}^{T}$$

$$\begin{array}{cc} subject\;to\; & g_{i}\left(x\right)\leq 0,i=1,\ldots ,p\\ \; & h_{j}\left(x\right)=0,j=1,\ldots ,q \end{array}$$

where $x=(x_{1},x_{2},\ldots ,x_{n})$ represents the decision variable vector, f(x) represents the *m*-objective functions, *m* represents the number of objective functions, and $g_{i}\left(x\right)\leq 0$ and $h_{j}\left(x\right)=0$ represent the *p* inequality and *q* equality constraints, respectively.

CMOPs involve optimizing multiple conflicting objectives subject to constraints that must be satisfied simultaneously, making it challenging to navigate towards the constrained Pareto front (CPF) [7,8] and obtain satisfactory solutions. Despite the numerous constrained multi-objective evolutionary algorithms (CMOEAs) proposed in the past two decades, only a few have been able to balance convergence, diversity, and feasibility, particularly when dealing with complex feasible regions. Therefore, further research and development of effective CMOEAs is urgently required.

To address this issue, this paper presents a novel algorithm, Constrained Multi-Objective Carnivorous Plant Algorithm (CMOCPA), which employs the carnivorous plant algorithm (CPA) [9] to solve CMOPs. To achieve a balance between convergence, diversity, and feasibility, CMOCPA utilizes multi-population and multi-stage methods with the secondary population that employs the ϵ-constrained handling method. Moreover, the algorithm introduces a cross-pollination method inspired by the trapping mechanism and pollination behavior of carnivorous plants, which improves the search efficiency of the algorithm. Additionally, a quasi-reflection learning mechanism is used for the growth process of carnivorous plants, while the quadratic interpolation method is introduced for the reproduction process of carnivorous plants. The experimental results on DC-DTLZ, FCP, and DASCMOP test suites show that CMOCPA outperforms existing state-of-the-art CMOEAs. Therefore, this paper makes significant contributions to the field of constrained multi-objective optimization. The contributions of this paper include:

- A new constrained multi-objective optimization algorithm, CMOCPA, is proposed. CMOCPA employs a two-population, two-stage method. The two populations, namely $Pop_{1}$, $Pop_{2}$, are employed for the original CMOP and the relaxed CMOP, respectively. $Pop_{1}$ concentrates on feasible solutions, while $Pop_{2}$ ignores all constraints in Stage 1 to quickly converge to the unconstrained Pareto front. In Stage 2, $Pop_{2}$ uses the ϵ-constrained handling method to guide the population back to CPF. The two populations are designed to help each other evolve, with $Pop_{1}$ providing guidance to $Pop_{2}$ in the search for feasible solutions, and $Pop_{2}$ providing diversity to $Pop_{1}$ by exploring the infeasible regions of the search space.
- Various novel mechanisms are introduced in CMOCPA, including a quasi-reflection learning mechanism in the growth process, quadratic interpolation in the reproduction process, and a cross-pollination method inspired by the trapping mechanism and pollination behavior of carnivorous plants. These mechanisms help the algorithm to improve the convergence speed, local exploitation ability, and ability to escape from local optima.

The remaining sections of this paper are organized as follows. In Section 2, a comprehensive overview of relevant concepts, including CPA and existing CMOEAs, is provided. In Section 3, the proposed algorithm, CMOCPA, is presented, and its two-population, two-phase strategy, as well as its mechanisms for growth process and reproduction process are described. Section 4 investigates the competitiveness of CMOCPA and compares it with the seven state-of-the-art CMOEAs on DC-DTLZ, FCP, and DASCMOP test suites. Finally, Section 5 gives the conclusion and future works.

## 2. Related Work

### 2.1. Carnivorous Plant Algorithm

CPA [9] is a plant-based algorithm [10,11] inspired by the trapping behavior of carnivorous plants. The algorithm simulates the attraction, trapping, growth, and reproduction process of carnivorous plants in the natural environment. CPA has the following steps:

The algorithm randomly initializes a population [12,13] containing *n* candidate solutions as the initial vector of carnivorous plants and prey. Following the initialization of the population, individuals are sorted in ascending order based on their fitness value (in the case of a minimization problem). The sorted population is divided into two categories: carnivorous plant and prey. The prey individuals are allocated to carnivorous plants based on their fitness values, forming the trapping range of the carnivorous plants. The carnivorous plants only trap within their designated range. This classification and grouping process emulates the way carnivorous plants attract and capture prey in their natural environment. After classification and grouping, the algorithm enters the growth process of carnivorous plants. The trapping of carnivorous plants is not always successful, so the algorithm introduces an attraction rate to simulate this process. Carnivorous plants will randomly select a prey within their attack range. When the random number is less than the attraction rate, the carnivorous plant hunts and digests the selected prey. On the contrary, when the random number is greater than the attraction rate, the prey will escape the trap of the carnivorous plant and will update its position. After the trapping process is complete, the carnivorous plants will enter the reproduction process. To avoid wasting computational resources caused by the generation of inferior offspring, CPA only selects carnivorous plants to reproduce. During the reproduction process, the optimal individual in the population guides the reproduction process of each individual. After the growth and reproduction, the newly generated individuals will be compared with the original population, and those with better fitness values will be selected to form the next generation population. The algorithm will repeat the process of grouping and classifying, growth, reproduction, and selection until the termination criteria are met.

CPA has been shown to be effective in solving a variety of optimization problems, including the traveling salesman problem [14,15] and heat-treated woods [16]. Its unique approach to simulating the trapping behavior of carnivorous plants provides a novel perspective on evolutionary algorithms and may inspire future research in this field.

### 2.2. Constraints of CMOPs

CMOPs are optimization problems with multiple conflicting objectives and constraints that must be satisfied simultaneously. For a CMOP, each solution has a degree of violation for each constraint. The constraint violation ($CV_{j}$) of a solution *x* at the *j*th constraint can be expressed as Equation (2).

(2) $$\begin{array}{c} CV_{j}=\left\{\begin{array}{ccc} \; & max(0,g_{j}\left(x\right)), & j=1,\ldots ,p\\ \; & max(0,|h_{j}\left(x\right)|-\eta ), & j=1,\ldots ,q \end{array}\right. \end{array}$$

where η is a very small value that can be used to relax equality constraints into inequality constraints. In general, the feasibility of a solution *x* depends on the sum of its violations at each constraint, as expressed in Equation (3).

(3) $$CV\left(x\right)=\sum_{j=1}^{q}CV_{j}\left(x\right)$$

When CV(x)=0, we refer to solution *x* as a feasible solution; otherwise, *x* is an infeasible solution. For a feasible solution, it must satisfy all of the problem’s constraints, including both equality and inequality constraints.

### 2.3. Existing CMOEAs with Constraint-Handling Technologies

Handling feasible and infeasible solutions resulting from constraints is a crucial aspect in solving CMOPs, for which there are two major categories of algorithms: feasibility-driven CMOEAs and infeasibility-assisted CMOEAs.

#### 2.3.1. Feasibility-Driven CMOEAs

Feasibility-driven CMOEAs prioritize feasible solutions over infeasible ones. For instance, MOSES [17] preserves feasible solutions and discards all infeasible ones, resulting in insufficient selection pressure for solutions in later stages, reducing the ability to converge to the true Pareto front. To increase the selection pressure, Deb et al. proposed the Constraint Domination Principle (CDP) in NSGA-II [18]. In CDP, constraints take precedence over objectives in the dominance relation to ensure that the solution is within the feasible region. CDP selects infeasible solutions based on constraint domination. Given two solutions $x_{a}$ and $x_{b}$, the $x_{a}$ constraint dominates $x_{b}$ if any of the following conditions are met:

1. Value $x_{a}$ is a feasible solution, and $x_{b}$ is an infeasible solution;
2. Both $x_{a}$ and $x_{b}$ are feasible solutions, and $x_{a}≺x_{b}$;
3. Both $x_{a}$ and $x_{b}$ are infeasible solutions, and $CV\left(x_{a}\right)<CV\left(x_{b}\right)$.

CDP selects infeasible solutions based on constraint domination, where the solution with fewer constraint violations dominates the one with more violations. When comparing two infeasible solutions, the one with fewer constraint violations is considered superior. When both solutions are feasible, the Pareto dominance relation is used to determine superiority. CDP has proven to be a powerful tool for handling constraints in CMOPs. By giving precedence to constraints over objectives in the dominance relation, CDP ensures that feasible solutions are always superior to infeasible ones. It has been adopted by many CMOEAs, including C-NSGA-III [18] and C-MOEA/D [19].

#### 2.3.2. Infeasibility-Assisted CMOEAs

CDP has limitations in handling certain types of constraints, such as nonlinear or logical operator-based constraints. To overcome these limitations, the ϵ-constraint handling method has been proposed as a popular infeasible auxiliary method. By introducing a relaxation factor, ϵ, the ϵ-constrained method allows for the retention of some infeasible solutions, thereby relaxing the comparison criteria of CDP. Given two solutions $x_{a}$ and $x_{b}$, $x_{a}$ dominates $x_{b}$ when any of the following conditions is met:

1. Value $x_{a}$ is feasible, and $x_{b}$ is infeasible;
2. Both $x_{a}$ and $x_{b}$ are infeasible, but $x_{a}$ violates fewer constraints than $x_{b}$;
3. Both $x_{a}$ and $x_{b}$ violate the same number of constraints, but $x_{a}$ has a smaller sum of constraint violation values than $x_{b}$.

Several CMOEAs based on the ϵ-constrained handling method have been developed to solve CMOPs, including the infeasible solutions diversity maintenance epsilon constraint handling method, an improved epsilon method with M2M [20] for solving imbalanced CMOPs with simultaneous convergence-hard and diversity-hard constraints, and ϵDE [21] using an adaptive ϵ-level control method and a combined fitness-violation epsilon constraint handling for differential evolution. Fan et al. [22] improved the ϵ-constrained handling method in MOEA/D for CMOPs with large infeasible regions. These methods have shown promise in addressing the challenges of solving CMOPs and improving the quality of obtained solutions.

The approach operates by defining a set of ϵ-constraints, which restrict the allowable range of objective function values for the infeasible solutions. The optimization problem is then transformed into multiple sub-problems, each with a different ϵ value, and these sub-problems are solved independently. The resulting set of solutions represents the Pareto front for each sub-problem, which can be combined to obtain the final Pareto front for the original problem.

Another method for handling constraints is the penalty function approach [23], which incorporates CVs into the fitness evaluation through a penalty function. In this approach, the fitness of an individual is calculated by adding a penalty term to the original objective function. The penalty term is proportional to the degree of constraint violation of the individual, encouraging the search to avoid infeasible solutions while allowing some infeasible solutions to be retained in the population for diversity. However, the penalty function approach can suffer from issues such as sensitivity to penalty parameters and premature convergence.

To improve the performance of penalty-function-based CMOEAs, some adaptive penalty methods [24] have been proposed. For instance, c-DPEA [25] proposes an adaptive penalty function to guide the population over infeasible regions, which allows for a more effective retention of some infeasible solutions in the population for diversity consideration. Similarly, in ShiP [26], the degree of shifting of infeasible solution positions is controlled based on the proportion of feasible solutions in the parent and child populations. The shifted solutions are then penalized according to their degree of CV violation.

Some CMOEAs convert CMOPs to unconstrained multi-objective optimization problems (UMOPs) by using overall constraint violation as an additional objective function. This approach has been employed in several algorithms, such as IDEA [27] and as presented by Peng et al. [28].

Other CMOEAs adopt a multi-stage method where the problem is tackled in two stages. Liu et al. [29] propose a two-stage algorithm that transforms CMOPs into constrained single-objective optimization problems in the first stage and then uses a specific CMOEA to find the final feasible solution in the second stage. Similarly, Fan et al. propose a push–pull search framework (PPS) [30] that first pushes the population to the unconstrained Pareto front using MOEA and then pulls the infeasible individuals towards the feasible region by a modified constraint-processing CMOEA.

Multi-population/archive methods co-evolve through collaboration between multiple populations/archives. For example, C-TAEA [31] proposes the use of two archives to balance convergence, diversity, and feasibility of the optimization process, where one archive pushes the population toward the Pareto front, while the other tends to maintain population diversity. BiCo [29] retains a master population and an archive to guide the search from both feasible and infeasible aspects. CCMO [32] is a weakly cooperative multi-population method in which one population solves CMOPs, while the other solves its unconstrained version.

## 3. The Proposed Algorithm

The effective handling of constraints is a critical issue when addressing CMOPs using CPA. In this paper, multi-population and multi-stage methods are combined with CPA to tackle this problem. The multi-population and multi-stage methods have been widely used in CMOEAs.

The multi-population method allows different populations to manage the archive and evolve separately for different purposes. For example, in a dual-population method, the main population is designated to prioritize feasibility, while the secondary population is used to converge quickly to the unconstrained Pareto front while ignoring the effects of infeasible regions caused by constraints. This allows the secondary population to provide information to the main population to help traverse the infeasible region. However, an important challenge with the multi-population method is that when the PF and CPF do not overlap fully, the secondary population’s convergence to the PF provides limited information to the main population. This limitation hampers the effectiveness of single multi-population CMOEAs in solving such problems, and the problem exacerbates as the distance between PF and CPF increases.

The multi-stage method divides the constraint-handling process into several stages. For instance, in a dual-stage method, constraints are not considered in the first stage, and the population quickly converges to the unconstrained Pareto front (PF). Then, in the second stage, constraints are taken into account using a constraint handling method such as the ϵ-constraint method. An important limitation of using a multi-stage approach alone is that when a population rapidly converges to the CPF without considering constraints in the first stage, it often overlooks critical information in the search space, such as feasible solutions with good convergence and diversity in the feasible region. This information could be essential in solving CMOPs.

This paper proposes a constrained multi-objective version of CPA, CMOCPA, which combines dual-population and dual-stage methods. In the first stage, the secondary population ignores constraints and converges quickly to the unconstrained PF while continuously providing information to the main population to help traverse the infeasible region. In the second stage, the secondary population uses the ϵ-constrained handling method to pull the population towards CPF with the help of the main population. The information obtained during this process can also be used to help the main population explore near the feasible region as it approaches the CPF from the PF. In addition, the paper proposes some improvement strategies for CMOCPA to enhance the search efficiency of the algorithm. Algorithm 1 presents the pseudocode for the proposed algorithm. Each part of the algorithm is described in detail as follows:
**Algorithm 1:**Procedure of CMOCPA
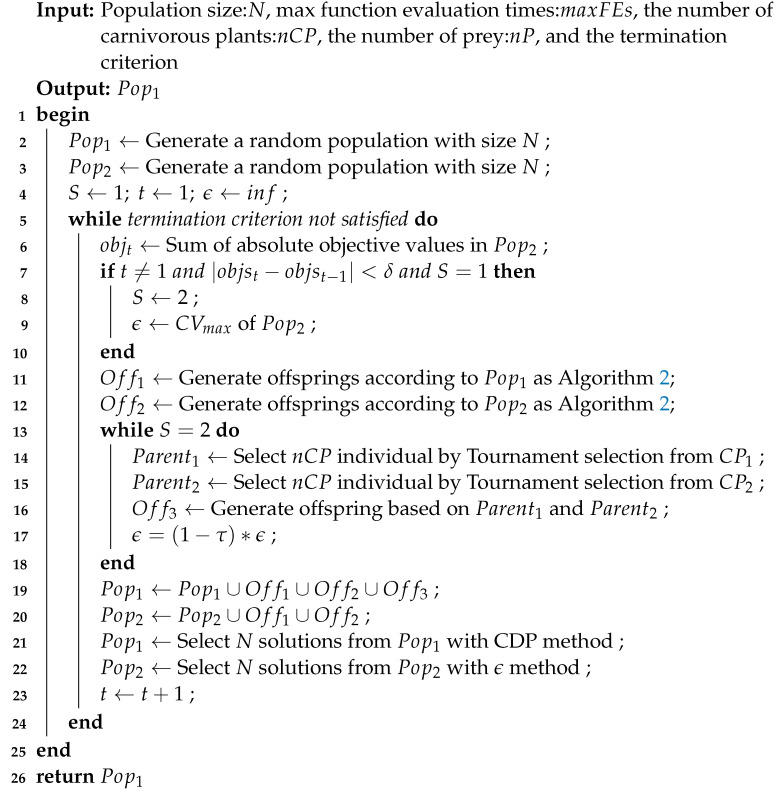


The initialization process is performed in lines 1–4, which involves parameter setting and population initialization.

The algorithmic parameters include:

- CPA parameters, such as growth_rate, reproduction_rate, and attraction_rate.
- The stage of the algorithm *S*. When S=1, the algorithm is in Stage 1, and $Pop_{2}$ is evolving without constraints. On the other hand, when S=2, the algorithm is in Stage 2, and $Pop_{2}$ is using the epsilon-constraints-handling method to ensure that its solutions are feasible with respect to the constraints.
- The initial value ϵ for $Pop_{2}$, which is set to be sufficiently large to ensure that all solutions in $Pop_{2}$ are feasible during Stage 1 when using the epsilon constraint method to select individuals.
- The threshold δ to determine whether individuals in $Pop_{2}$ have reached a stable state and whether the algorithm should move from Stage 1 to Stage 2.

Population Initialization: The algorithm first generates two populations randomly for the initial population. In the population initialization process, the algorithm randomly initializes two population ($Pop_{1}$ and $Pop_{2}$) containing np candidate solutions as the initial vector of carnivorous plants and prey. The population is described by a matrix, as shown in Equation (4).

(4) $$pop=\left[\begin{array}{cccc} \; & x_{1,1} & \ldots & x_{1,d}\\ \; & \vdots & \ddots & \vdots \\ \; & x_{n,1} & \ldots & x_{n,d} \end{array}\right]$$

where *d* is the number of decision variables, and *n* is the number of individuals in the population. Each individual in the population is initialized using random initialization based on a uniformly distributed random number in the range of [0,1], as shown in Equation (5).

(5) $$x_{i,j}=lb_{i,j}+rand*\left(ub_{i,j}-lb_{i,j}\right)$$

where lb and ub are the lower and upper bounds of the search domain, respectively, $x_{i,j}$ is the *j*th decision variable of the *i*th candidate solution, i∈[1,2,…,n], j∈[1,2,…,d], and rand is a uniformly distributed random number in the range of [0,1].

During Stage 1, the value of ϵ is retained at its initial setting to emphasize the diversity and convergence of solutions during the selection process of $Pop_{2}$ using the epsilon-constrained method. The difference between the absolute sum of CVs in $Pop_{2}$ of the current and preceding generations is calculated. If this value is lower than δ, $Pop_{2}$ is considered to have achieved convergence to the unconstrained Pareto front. At this point, the maximum constraint violation value in $Pop_{2}$ is used to set ϵ, and S=2 is set to enter Stage 2.

The offspring-generation process is described in Lines 11 and 12, and a detailed process is presented in Algorithm 2. The algorithm utilizes the CPA framework to optimize solutions and is mainly divided into the following processes:
**Algorithm 2:** Generate offspring of CMOCPA
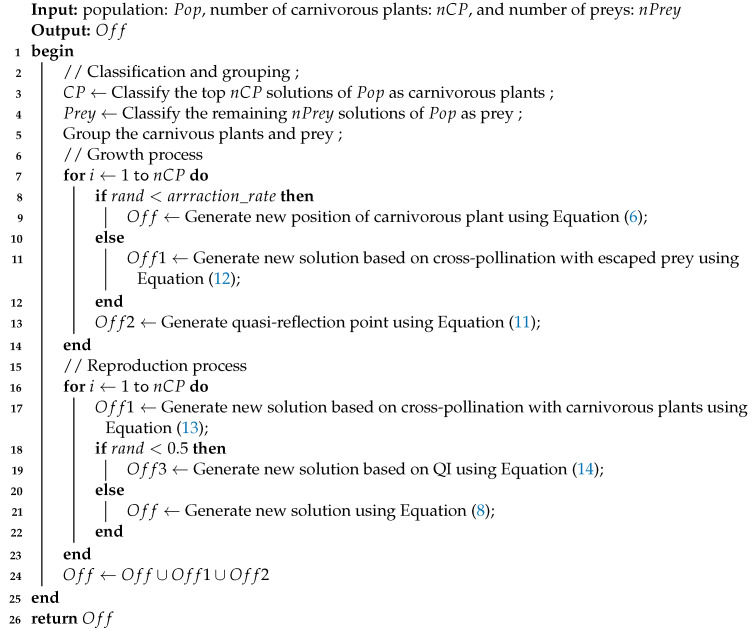

- Classification and grouping: The algorithm divides the population into two categories: carnivorous plants and prey. The population is further divided into several groups, with the best prey assigned to the best carnivorous plant based on fitness value. Subsequent prey are then assigned to subsequent carnivorous plants in order of fitness value until all preys are assigned. Carnivorous plants only prey within their trapping range. Fitness calculation of the CMOPs version is carried out in the same way as in SPEA2 [33];
- The growth process: After classification and grouping, the carnivorous plant selects a prey randomly in its trapping range. When the random number is less than the attraction rate, the prey is trapped and digested by the carnivorous plant, and the plant’s position is updated according to Equation (6).
  (6) $$NewCP_{i,j}=growth*CP_{i,j}+\left(1-growth\right)*Prey_{v,j}$$
  where $CP_{i,j}$ is the *j*th decision variable for the *i*th level of carnivorous plants, $Prey_{v,j}$ is the jth-dimension decision variable for a randomly selected prey, the growth rate is a predefined value, and rand is a randomly selected value from the range [0,1]. The magnitude of carnivorous plant growth is expressed as Growth and is calculated using Equation (7).
  (7) $$growth=growth\_rate*rand_{i,j}$$
  If the random number is less than the attraction rate, the selected prey will not be trapped. This prey is considered a pollinator, helping the carnivorous plant to complete cross-pollination. Once an offspring is generated, the quasi-reflection learning mechanism is used to generate a quasi-reflection point between the offspring and the parent. These offspring and quasi-reflection points are added to the environmental selection to improve the population diversity and search efficiency of the algorithm. The quasi-reflection and cross-pollination are described in detail in Section 3.1 and Section 3.2, respectively.
- The reproduction process: The reproduction process begins once carnivorous plants complete the trapping process. Only carnivorous plants are eligible for reproduction to save computing resources. Since the original reproduction method is always guided by the optimal individual, the algorithm’s diversity gradually decreases as individuals become closer to the optimal solution. To address this issue, a cross-pollination method based on Lévy flight is proposed to increase the algorithm’s diversity and exploratory ability, thus avoiding getting trapped in local optima. The original reproduction method of CPA is treated as self-pollination. With a certain chance, carnivorous plants generate offspring by self-pollination as shown in Equation (8).
  (8) $$NewCP_{i,j}=CP_{1,j}+Reproduction\_rate*rand_{i,j}*mate_{i,j}$$
  where $CP_{1,j}$ is the best solution, $CP_{v,j}$ is a randomly selected carnivorous plant, and the reproduction rate is a predetermined utilization value. This process is repeated for each of the nCPlant carnivorous plants. The value mate is selected based on the fitness values of the random carnivorous plant and the best carnivorous plant to ensure that the algorithm approaches the current best carnivorous plant; mate is calculated as shown in Equation (9).
  (9) $$mate_{i,j}=\left\{\begin{array}{ccc} \; & CP_{v,j}-CP_{i,j}, & f\left(CP_{i}\right)>f\left(CP_{v}\right)\\ \; & CP_{i,j}-CP_{v,j}, & f\left(CP_{i}\right)<f\left(CP_{v}\right) \end{array}\right.,i\neq v\neq 1$$
  In addition, a quadratic interpolation method is introduced, where extreme points fitted by curve-fitting replace the current offspring with a certain probability. The cross-pollination behavior and the reproduction process based on quadratic interpolation will be described in detail in Section 3.2 and Section 3.3. The newly generated individuals are then merged into the original population for environmental selection.

In Stage 2 of the algorithm, the goal is to move from the unconstrained Pareto front to the constrained Pareto front while maintaining good diversity and convergence. To accomplish this, the algorithm gradually reduces the value of epsilon, which allows $Pop_{2}$ to prioritize feasibility without sacrificing the quality of solutions. In addition to this, individuals from different populations are considered to originate from different environments, and cross-environment pollination is used to generate offspring between $Pop_{1}$ and $Pop_{2}$. This helps to explore the feasible area near Population 1 and maintain diversity in $Pop_{2}$. By combining these two techniques, the algorithm is able to effectively balance the need for feasibility and quality of solutions while maintaining diversity and exploring the search space.

Similar to most existing CMOEAs, CMOCPA employs an elite strategy to select the best solutions from the combined populations to be carried over to the next generation through environmental selection. This strategy ensures that high-quality solutions are preserved and propagated in the population, while low-quality solutions are removed. This helps to improve the overall quality of the population over time and increase the likelihood of finding optimal or near-optimal solutions. CDP is used to select feasible solutions from $Pop_{1}$, while the ϵ-constraint handling method is used to select individuals from $Pop_{2}$ that satisfy the constraints. This approach ensures that $Pop_{1}$ continues to focus on feasibility, while $Pop_{2}$ gradually shifts its focus towards feasible solutions as the value of epsilon decreases. Throughout the optimization process, $Pop_{1}$ receives information about feasible solutions from $Pop_{2}$ to accelerate convergence and improve the overall performance of the algorithm.

### 3.1. Improved Growth Process Based on Quasi-Reflection Learning

During the growth process of CMOCPA, the algorithm is typically oriented towards the optimal individual to accelerate convergence. However, this strategy often leads to a decrease in population diversity, making it difficult for the algorithm to escape local optima. To address these problems, quasi-reflection-based learning is introduced to enhance population diversity and the algorithm’s probing ability to help the population escape local optima.

Quasi-reflection-based learning (QRBL) [34,35] is a variant of Opposition-based learning (OBL) [36], which is a search strategy based on local search. QRBL works by first selecting a candidate individual as the current best solution, and then generating a new candidate solution through a reflection operation. The quasi-reflection number and quasi-reflection point are used to generate the new solution as shown in Equations (10) and (11).

**Definition** **1.** *Quasi-reflection number.*

(10) $$x^{qr}=rand\left(\frac{lb+ub}{2},x\right)$$

*where lb and ub are the lower and upper bounds of the search space, respectively; x is a real number in [lb,ub].*

**Definition** **2.** *Quasi-reflection point.*

*Suppose the solution $x=[x_{1},x_{2},\ldots ,x_{n}]$, where $x_{1},x_{2},\ldots ,x_{n}\in R$ and $x_{j}\in \left[lb_{j},ub_{j}\right]$. The quasi-opposite number can be extended to d-dimensional space by using Equation (11).*

(11) $$x_{j}^{qr}=rand\left(\frac{lb_{j}+ub_{j}}{2},x_{j}\right)$$

During the growth process of CPA, when an offspring is generated, quasi-reflection learning is used to search in the vicinity of the current candidate solution in the search space, generate a solution between the current solution and the offspring, and add this solution to the environmental selection.

### 3.2. Cross-Pollination Based on Lévy’s Flight

Inspired by the distinctive trapping and pollination behaviors of carnivorous plants, the breeding process of the CPA algorithm has been improved. Carnivorous plants rely on insects for pollination to support their growth and reproduction, but excessive consumption of insects can negatively impact their reproductive success. To address this, certain carnivorous plants have evolved mechanisms that spare some insects, such as producing flowers and traps at different times or locations or using sticky hairs that do not harm pollinators. These insects can assist in pollinating the plants and may exhibit Lévy flight behavior, which is beneficial for optimizing search algorithms.

The ability of prey to escape traps can aid carnivorous plants in achieving global pollination. Such “escaped prey” can assist in pollinating carnivorous plants and may exhibit a preference for certain individuals, such as the most optimal individual $CP_{1}$, which appears more attractive to pollinators due to characteristics such as brighter color and so on. In addition, insects may exhibit Lévy flight characteristics. To simulate the Lévy flight behavior that pollinators may exhibit during pollination, the Lévy flight strategy has been introduced into cross-pollination as shown in Equation (12).

(12) $$Off=CP_{i}+Levy\left(\lambda \right)⊕\left(CP_{1}-Prey_{v}\right)$$

where $CP_{i}$ represents the carnivorous plant, and $Prey_{v}$ is the insect that has escaped the trap within the attack range of $CP_{i}$; ⊕ is the dot product.

Cross-pollination is an important process in the reproduction of plants, and it can occur through different mechanisms, including insects, wind, and water. The original method of reproduction is considered to be self-pollination, but to improve the algorithm’s efficiency and accuracy, cross-pollination assisted by wind or water has been taken into account.

Equation (13) shows the formula for cross-pollination without insects. It involves selecting two different carnivorous plants randomly and generating a new offspring through a weighted-average Lévy. This process allows the algorithm to explore different regions of the search space and potentially find better solutions.

(13) $$Off=CP_{i}+Levy\left(\lambda \right)⊕\left(CP_{1}-Prey_{v}\right)$$

### 3.3. Improved Reproduction Process Based on Quadratic Interpolation Method

CPA selects the individual with the best fitness value for reproduction to guide other individuals towards the best position, focusing on the best solution. However, when applied to constrained multi-objective optimization problems, the algorithm’s reproduction behavior suffers from defects such as slow search speed and a lack of local detection ability. To address these defects, the quadratic interpolation (QI) [37,38] operator is introduced to improve the algorithm’s local exploitation ability and convergence speed.

The QI operator is a local search operator that constructs a quadratic polynomial similar to the objective function using the function values of the objective function at three different points. It then uses the extreme points of this quadratic polynomial as the approximate extreme points to approximate the objective function. The QI operator is commonly used in many optimization algorithms to develop better solutions for specific populations. The QI operator can rapidly approximate the optimal solution when the population is located near the global solution, which is essential for improving the local exploitation of CMOCPA. The QI operator updates the position of carnivorous plants using Equation (14).

(14) $$X_{i}=\frac{\left(CP_{1}^{2}-CP_{r1}^{2}\right)*f\left(CP_{r2}\right)+\left(CP_{r2}^{2}-CP_{1}^{2}\right)*f\left(CP_{r1}\right)+\left(CP_{r1}^{2}-CP_{r2}^{2}\right)*f\left(CP_{1}\right)}{2*[\left(CP_{1}-CP_{r1}\right)*f\left(CP_{r2}\right)+\left(CP_{r2}-CP_{1}\right)*f\left(CP_{r1}\right)+\left(CP_{r1}-CP_{r2}\right)*f\left(CP_{1}\right)]}$$

where $f(CP_{1})$, $f(CP_{r1})$, and $f(CP_{r2})$ are the fitnesses of three different carnivorous plants $CP_{1}$, $CP_{r1}$, and $CP_{r2}$, respectively. $CP_{1}$ is the individual with the best fitness value among carnivorous plants, and $CP_{r1}$ and $CP_{r2}$ are different carnivorous plants.

By using the QI operator, the position of the carnivorous plant can be updated more efficiently, which improves the local exploitation ability of the algorithm. This is crucial for CMOPs, where diversity of solutions is not guaranteed. The QI operator’s ability to rapidly approximate the optimal solution helps to improve the algorithm’s convergence speed and search ability, thus leading to better results.

## 4. Simulation Experiments and Results Analysis

In this section, Section 4.1 gives the experimental setup, including the test suite used for testing and the state-of-the-art CMOEAs used for comparison, and describes the performance metrics used. Section 4.2 gives the experimental results and analysis.

### 4.1. Experimental Settings

To show the superiority of CMOCPA, seven CMOEAs were selected: BiCo [29], C-TAEA [31], CAEAD [39], ICMA [40], PPS [30], ToP [41], and Trip [42]. To validate the performance of CMOCPA on ZDT [43], DTLZ [44], and RWMOPs [45], seven algorithms were used for comparison, including MOEAD [46], eMOEA [47], MOPSO [48], NSGAII [18], SPEA2 [33], KnEA [49], and GrEA [50]. The default parameters in PlatEMO v4.0 [51] were used where there is no particular explanation.

#### 4.1.1. Benchmark Problems

DC-DTLZ [31], FCP [40], DASCMOP [52], ZDT, DTLZ, and RWMOPs are benchmark problems used to evaluate the performance of constrained multi-objective optimization algorithms, including CMOCPA.

DC-DTLZ is a variant of C-DTLZ [53] in which the constraint conditions are dynamically changing. In DC-DTLZ, the number and type of constraint conditions vary over time, which increases the complexity and challenge of the problem and makes it closer to practical problems. FCP is a set of fixed-budget constrained optimization problems that are designed to evaluate the performance of constrained optimization algorithms under a limited computational budget. DASCMOP is a class of dynamic and adaptive multi-objective optimization problems that test the ability of algorithms to track time-varying Pareto fronts.

By evaluating the performance of CMOCPA on these benchmark problems, one can assess the algorithm’s ability to find high-quality solutions for constrained multi-objective optimization problems and compare its performance to other state-of-the-art algorithms. The following parameters are typically set for each benchmark:

Number of Runs: Each algorithm is run 30 times independently for each test instance. For all benchmark problems, the population size N=100. Their objective number *M*, decision vector *D*, and number of function evaluations FEs are as follows:

- For all DC-DTLZ: FEs=100,000, for DC1-DTLZ1, DC2-DTLZ1, and DC3-DTLZ1, D is set to 7; D is set to 12 for the remaining DC-DTLZ problems.
- For all FCP problems, FEs=200,000; for other parameters refer to ICMA [40];
- For all DASCMOP problems, D=30, FEs=300,000; for DASCMOP1-DASCMOP6, M=2; for DASCMOP7-DASCMOP9, M=3.
- For ZDT and DTLZ, FEs=50,000.
- For RWMOPs, all parameters are the same as in [45].

#### 4.1.2. Genetic Operators and Parameter Settings

The pull stage of PPS, ToP, and CAEAD adopt DE [54] while other CMOEAs in comparison adopt GA [55]. The experimental parameters are listed as follows:

- Simulated binary crossover (SBX): $p_{c}=1$, $\eta_{c}=20$;
- Polynomial mutation (PM): $p_{m}=1/D$, $\eta_{m}=20$;
- DE operators: CR=1.0, F=0.5;
- ToP parameters: $p_{f}=1/3$, δ=0.2;
- PPS parameters: $T_{c}=800$, α=0.9, τ=0.1, cp=2, l=20;
- MOEAD parameters: T=N/10;
- eMOEAD parameters: ϵ=0.06;
- MOPSO parameters: div=10;
- KnEA parameters: r=0.5;
- GrEA parameters: div=[0,45,15,10,9,9,8,8,10,12];
- CMOCPA parameters: attraction_rate=0.8, reproduction_rate=1.8, growth_rate=2;

#### 4.1.3. Performance Metrics

Inverted Generational Distance (IGD) [56] is a comprehensive performance evaluation metric. It is improved from the General Distance (GD) [57]. By calculating the minimum distance sum between each individual on the real PF and the set of individuals obtained by the algorithm, the convergence and diversity performance of the algorithm can be evaluated simultaneously. The smaller the IGD, the better the integrated performance, including convergence and diversity. The *IGD* value is calculated as shown in Equation (15).

(15) $$IGD\left(P,Q\right)=\frac{\sum_{v\in P}d\left(v,Q\right)}{\left|P\right|}$$

where *P* is the set of points uniformly distributed on the real PF, *p* is the number of individuals in the set of points distributed on the real PF, and Q is the optimal Pareto optimal solution set obtained by the algorithm; d(v,Q) is the minimum Euclidean distance from the individual *v* in p to the overall *Q*. Thus, IGD evaluates the comprehensive performance of the algorithm by computing the average of the minimum Euclidean distances between the set of points on the real PF and the obtained Pareto.

Hypervolume (HV) [58] is a commonly used evaluation metric that reflects how close the set of non-dominated solutions obtained by CMOEA is to the true PF. The *HV* of the solution set *S* is shown in Equation (16).

(16) $$HV\left(S\right)=VOL\left(\underset{i\in P}{⋃}\left\lceil f_{1}\left(i\right),z_{1}^{r}\right\rceil \right)$$

where VOL(.) is the Lebesgue measure, *m* denotes the number of objectives, and $z^{r}=\left(z_{1}^{r},\ldots ,z_{m}^{r}\right)$ is a user-defined reference point in the objective space. The bigger the HV value, the better the performance of the algorithm.

### 4.2. Experimental Results

In this section, the performance of CMOCPA is compared with seven other CMOEAs on three test suites using two widely used metrics, namely, IGD and HV, to evaluate their performance. The Wilcoxon rank sum test [59] at 0.05 significance level is performed between CMOCPA and its peer algorithms on the IGD and HV results. CMOCPA is compared with each competitor in a paired comparison. The symbols “+”, “−”, or “≈” indicate whether the corresponding competitor is superior to, inferior to, or equivalent to CMOCPA, respectively. Additionally, the best metric values for each problem are highlighted in yellow in each table. Appendix A shows the population with median IGD value among 30 runs obtained by MOEAD, eMOEA, MOPSO, NSGAII, SPEA2, KnEA, GrEA and CMOCPA on each problem and other supplementary data.

#### 4.2.1. Result on DC-DTLZ Benchmark Problems

DC-DTLZ is a challenging multi-objective optimization problem that involves dynamic constraints. In this problem, constraints can change dynamically, which increases the complexity of the optimization process. The objective function structure in DC-DTLZ is similar to that of the DTLZ function, with the main difference being the dynamic nature of the constraints. New constraints can be added or removed at each iteration, making the problem even more challenging.

The experimental results for the DC-DTLZ problem, presented in Table 1 and Table 2, demonstrate that the proposed algorithm in this paper outperforms the comparison algorithms. Analysis of the IGD metrics shows that CMOCPA achieves the best results in five out of the six problems. When compared to BiCo, the proposed algorithm outperforms BiCo in three results, with the remaining result showing no significant difference. Compared to other algorithms not included in BiCo, CMOCPA significantly outperforms these algorithms in all problems.

Regarding the HV metrics, the results indicate that the proposed algorithm has a significant advantage over BiCo in three out of six problems, lags behind in two results, and achieves similar results in one. This suggests that CMOCPA has a slight advantage over BiCo in terms of HV metrics. Furthermore, compared to other algorithms, apart from BiCo, CMOCPA clearly outperforms them in all problems based on HV metrics.

The experimental results based on IGD and HV metrics demonstrate that the proposed CMOCPA algorithm performs well in terms of diversity and convergence, outperforming BiCo in some cases. Compared to other algorithms besides BiCo, CMOCPA shows a significant advantage in solving DC-DTLZ problems. These results suggest that CMOCPA is a competitive algorithm for solving DC-DTLZ problems.

#### 4.2.2. Result on FCP Benchmark Problems

Table 3 and Table 4 demonstrate that CMOCPA exhibits superior performance to the other seven algorithms on the FCP test suite, which comprises CMOPs featuring deceptive constraints. Due to the non-monotonic and randomly generated nature of constraint violations in FCP problems, discovering the CPF presents a formidable challenge to algorithms.

**Figure 1:**
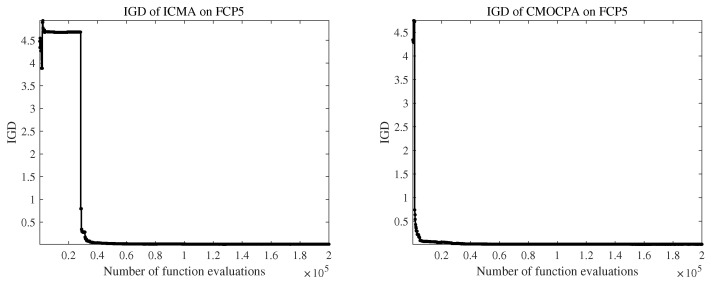
IGD convergence of ICMA and CMOCPA on FCP5.

Regarding FCP1–FCP4, only ICMA and CMOCPA were capable of identifying the CPF, whereas other algorithms quickly converged to locally infeasible regions. ICMA assumes the entire search space as a promising region, resulting in some evaluations being wasted due to the oversized objective function upfront. In contrast, CMOCPA employs a two-population, two-stage method to simultaneously evolve feasible and infeasible solutions. Additionally, CMOCPA leverages the Lévy flight strategy to perturb the populations and enhance the algorithm’s ability to escape local optima. As a result, CMOCPA discovers the feasible region more rapidly than ICMA, as demonstrated in Figure 1.

With respect to FCP5, all algorithms except ICMA and CMOCPA found partial CPFs. For example, C-TAEA incorporates two archives: DA and CA. However, DA disregards constraints and tends to identify small convergence values below the CPF while neglecting the feasible region above. Hence, such algorithms can only detect fragments of the CPF. In contrast, ICMA and CMOCPA discovered the entire convergence region. CMOCPA incorporates search strategies that enhance its capabilities and allow for a more-refined search process. Notably, CMOCPA exhibits superior diversity and convergence compared to ICMA, setting it apart from the latter.

#### 4.2.3. Result on DASCMOP Benchmark Problems

DASCMOP is a dynamic multi-objective optimization problem that takes into account the impact of dynamics and uncertainty in constrained multi-objective optimization problems. It requires identifying optimal solutions for a multi-objective function, the constraints of which vary with time. Furthermore, each objective function includes a stochastic component, adding to the complexity and uncertainty of the problem.

Table 5 and Table 6 present the IGD and HV results, respectively, of CMOCPA and other comparable algorithms for the DASCMOP problem. CMOCPA outperforms other algorithms in terms of IGD metrics, with six out of nine test problems achieving optimal results. The IGD metric is a measure of convergence and diversity, and CMOCPA’s multi-population and multi-stage strategy allows Pop2 to provide a more diverse solution than Pop1, facilitating faster coverage of the feasible region when dealing with the DASCMOP problem.

In terms of the HV metric, CMOCPA achieves the best performance in four out of nine problems and is not statistically significantly different from ICMA but outperforms the other compared algorithms. However, comparing the performance of optimization algorithms using HV values can be misleading since HV is sensitive to the distribution and scaling of the Pareto-optimal front. In contrast, IGD measures the distance between the true Pareto-optimal front and the approximation obtained by the optimization algorithm. The superior IGD results of CMOCPA demonstrate its capability to handle dynamic and stochastic multi-objective optimization problems with constraints, outperforming other state-of-the-art algorithms in terms of both convergence and diversity.

#### 4.2.4. Result on ZDT and DTLZ Benchmark Problems

ZDT and DTLZ offer a total of twelve test functions that serve as standard benchmarks for evaluating the performance of multi-objective optimization algorithms. ZDT comprises five test functions, namely ZDT1, ZDT2, ZDT3, ZDT4, and ZDT6, which are distinguished by varying numbers and types of objective functions, including both linear and nonlinear ones. In contrast, DTLZ offers seven test functions, namely DTLZ1, DTLZ2, DTLZ3, DTLZ4, DTLZ5, DTLZ6, and DTLZ7, which are similar to ZDT functions in having multiple objective functions and diverse features. However, the DTLZ functions are more complex, featuring more objective functions and nonlinear properties than the ZDT functions.

Table 7 and Table 8 present the IGD and HV results, respectively, of CMOCPA and other comparable algorithms for ZDT and DTLZ problems. CMOCPA outperforms other algorithms in terms of IGD metrics, with eight out of twelve test problems achieving optimal results. In terms of the HV metric, CMOCPA achieves the best performance in six out of twelve problems.

Compared to SPEA2, CMOCPA performs significantly worse on two problems, significantly better on eight problems, and similarly on two problems. The algorithm has two populations evolving simultaneously, which may result in wasted evaluation time when the two populations are close to each other. This may result in suboptimal convergence in later stages. Analysis of the HV metrics reveals that CMOCPA performs well on the majority of problems, with only a few problems demonstrating weaker performance compared to SPEA2.

Overall, CMOCPA is effective on ZDT and DTLZ test problems, indicating that it can be applied beyond CMOPs and is also competitive for MOPs.

#### 4.2.5. Result on RWMOPs Benchmark Problems

RWMOPs are a set of benchmark problems widely used in the field of multi-objective optimization. These problems are designed to simulate real-world scenarios, taking into account various complexities and constraints that are commonly encountered in practical applications. The problems included in the RWMOPs cover a wide range of engineering and scientific disciplines, reflecting the diversity of real-world optimization problems.

(1) Results on mechanical design problems (RWMOP1-RWMOP21)

Mechanical design problems focus on optimizing the design of mechanical components or systems, taking into account factors such as strength, durability, weight, and cost. Table 9 and Table 10 present the IGD and HV values for CMOCPA and seven other algorithms in solving mechanical design problems. Table 9 shows that CMOCPA obtained eleven optimal solutions, followed by eMOEA with three, NSGAII with six, and KnEA with two out of twenty-two problems. Notably, RWMOP8, RWMOP13, RWMOP19, and RWMOP20 are multimodal problems, which are difficult to converge to the PF. However, CMOCPA performed well on these problems. In terms of IGD, CMOCPA outperformed NSGAII on ten problems, had similar results on seven, and performed worse on four. As for HV, CMOCPA outperformed NSGAII on eight problems, had similar results on four, and performed worse on nine. Overall, CMOCPA and NSGAII had similar performance on mechanical design problems.

(2) Results on chemical engineering problems (RWMOP22-RWMOP24)

Chemical engineering problems typically involve optimizing chemical processes such as distillation or chemical reactions with objectives such as maximizing yield or minimizing energy consumption. Table 11 and Table 12 present the IGD and HV values for CMOCPA and other algorithms in solving chemical engineering problems. RWMOP22, a realistic problem with many local optimal solutions, poses a difficult convergence challenge. However, CMOCPA’s Levy’s individual perturbation mechanism allows it to escape local optima effectively. For RWMOP23, which has a very decentralized solution, NSGAII, KnEA, and CMOCPA performed similarly. For RWMOP24, which is similar to RWMOP22, CMOCPA is the only algorithm that could find feasible solutions.

(3) Results on process, design, and synthesis problems (RWMOP25-RWMOP29)

Process design and synthesis problems aim to optimize the design of complex industrial processes, taking into account factors such as equipment selection, scheduling, and resource allocation. Table 13 and Table 14 present the IGD and HV values for CMOCPA and seven other algorithms in solving process, design, and synthesis problems. These problems pose several optimization challenges, such as non-separability, multimodality, bias, deception, many-to-one mapping, and PF shape combinations. CMOCPA obtained optimal results in terms of IGD metrics for all problems, including the difficult-to-converge RWMOP28, for which other algorithms were trapped in local PFs. For the relatively simple RWMOP25 and RWMOP27, CMOCPA provided well-distributed solutions compared to other optimizers. Additionally, CMOCPA demonstrated good convergence and diversity in RWMOP26, whereas other algorithms, except CMOCPA, struggled to obtain well-converged solutions.

(4) Results on power electronics problems (RWMOP30-RWMOP35)

Power electronics problems are another important category of RWMOPs, focusing on the optimization of electrical systems that involve the conversion and control of power. These problems often involve a wide range of objectives, such as maximizing efficiency, minimizing losses, and ensuring stable operation under varying conditions. Table 15 and Table 16 present the IGD and HV metrics for CMOCPA and other algorithms in solving power electronic problems, which have many inequality constraints. RWMOP30-RWMOP33 have twenty-four inequality constraints, while RWMOP34 and RWMOP35 have twenty-nine inequality constraints, making it difficult to converge to the true PF. CMOCPA outperformed other algorithms in four out of the six problems.

(5) Results on power-system optimization problems (RWMOP36-RWMOP50)

Power-system optimization problems aim to optimize the operation of large-scale electrical grids, taking into account factors such as demand, generation capacity, and transmission constraints. Table 17 and Table 18 display the IGD and HV metrics for these problems, which are known for their numerous equality constraints and are challenging for most algorithms to obtain optimal solutions. The results indicate that CMOCPA achieved the best performance on two out of the fifteen problems, namely RWMOP49 and RWMOP50, while almost all other algorithms struggled to find feasible solutions for most of the problems. Notably, NSGAII, KnEA, and CMOCPA were able to obtain feasible solutions on RWMOP50, whereas only CMOCPA was able to find feasible solutions on RWMOP49.

In summary, the results indicate that CMOCPA is a promising optimization algorithm for resolving RWMOPs, particularly for problems with complex constraints and difficult-to-converge PFs.

## 5. Conclusions and Future Work

As a plant-based algorithm, CPA has demonstrated strong performance in solving single-objective optimization problems. To extend its capabilities to CMOPs, this paper introduces a new algorithm called CMOCPA. The algorithm adopts a multi-population and multi-stage framework that combines the Lévy flight-based cross-pollination of carnivorous plants with the quasi-reflection learning mechanism. These enhancements improve the algorithm’s ability to jump out of local optima, increase convergence efficiency and accuracy, and make local search more effective using quadratic interpolation methods.

To verify the stability, convergence accuracy, and optimality finding capability of CMOCPA, experiments were conducted on six test suites, including DC-DTLZ, FCP, DASCMOP, ZDT, DTLZ, and RWMOPs with eighty-two test problems. The experimental results show that CMOCPA performs well in terms of diversity and convergence, especially on the FCP problem, where it outperforms all other algorithms. CMOCPA also achieves better results for other problems, indicating that the proposed strategy are effective in improving the optimization performance of CMOCPA.

Given the effectiveness of CPA in solving CMOPs, future work will explore further applications of plant-based algorithms in this area.

## Figures and Tables

**Table 1:** Mean and standard deviation of IGD values on DC-DTLZ; ’NAN’ indicates that no feasible solution was found; best result is highlighted in yellow.

Problem	BiCo	CTAEA	CAEAD	ICMA	PPS	ToP	TriP	CMOCPA
DC1_DTLZ1	1.1584e-2 (1.20e-4) −	1.5158e-2 (2.56e-4) −	1.0618e-1 (1.55e-1) −	1.1893e-2 (1.60e-4) −	2.8409e-2 (9.10e-3) −	2.1664e-2 (6.14e-3) −	1.2794e-2 (2.60e-4) −	1.1488e-2 (2.06e-4)
DC1_DTLZ3	3.4686e-2 (7.70e-4) ≈	4.3288e-2 (1.50e-3) −	1.0197e+0 (1.29e+0) −	5.3560e-2 (3.73e-2) −	3.7055e-1 (2.35e-1) −	8.9654e-1 (1.11e+0) −	3.6929e-2 (7.67e-4) −	3.4627e-2 (1.39e-3)
DC2_DTLZ1	5.2700e-2 (6.42e-2) ≈	2.3224e-2 (1.87e-4) −	7.7302e-2 (6.92e-2) −	5.5734e-2 (6.54e-2) −	5.1453e-2 (5.42e-2) −	NaN (NaN)	2.2525e-2 (6.59e-4) −	2.0804e-2 (4.65e-4)
DC2_DTLZ3	5.6324e-1 (1.81e-3) −	1.4721e-1 (1.85e-1) −	4.0076e-1 (2.61e-1) −	2.6150e-1 (2.79e-1) −	3.4354e-1 (2.54e-1) −	NaN (NaN)	1.7668e-1 (1.93e-1) −	5.4735e-2 (1.00e-3)
DC3_DTLZ1	2.9754e-2 (5.59e-2) −	9.3533e-3 (2.12e-4) −	9.8043e-1 (6.23e-1) −	7.0463e-3 (1.35e-4) −	3.1353e-1 (3.70e-1) −	2.1104e+0 (2.31e+0) −	7.6022e-3 (3.51e-4) −	6.8449e-3 (8.80e-5)
DC3_DTLZ3	9.4436e-1 (4.65e-1) −	2.8206e-2 (8.64e-3) +	4.5099e+0 (3.57e+0) −	9.4954e-1 (4.70e-1) −	2.2421e+0 (2.11e+0) −	8.3246e+0 (4.28e+0) −	2.7967e-1 (2.49e-1) −	3.0185e-2 (3.42e-2)
+/−/≈	0/4/2	1/5/0	0/6/0	0/6/0	0/6/0	0/4/0	0/6/0	

**Table 2:** Mean and standard deviation of HV values on DC-DTLZ; ’NAN’ indicates that no feasible solution was found; best result is highlighted in yellow.

Problem	BiCo	CTAEA	CAEAD	ICMA	PPS	ToP	TriP	CMOCPA
DC1_DTLZ1	6.3234e-1 (6.43e-4) +	6.2733e-1 (5.11e-4) −	4.2919e-1 (2.23e-1) −	6.2323e-1 (2.02e-3) −	5.8118e-1 (2.36e-2) −	5.8213e-1 (2.38e-2) −	6.2773e-1 (1.69e-3) −	6.3073e-1 (1.22e-3)
DC1_DTLZ3	4.7345e-1 (1.22e-3) +	4.6238e-1 (1.95e-3) −	1.1362e-1 (1.49e-1) −	4.2699e-1 (7.22e-2) −	2.6423e-1 (1.53e-1) −	1.3585e-1 (1.80e-1) −	4.6865e-1 (2.40e-3) −	4.6982e-1 (3.62e-3)
DC2_DTLZ1	7.5947e-1 (1.62e-1) ≈	8.3810e-1 (4.37e-4) −	6.8824e-1 (1.82e-1) −	7.5158e-1 (1.66e-1) −	7.4660e-1 (1.42e-1) −	NaN (NaN)	8.3582e-1 (2.75e-3) −	8.3958e-1 (1.71e-3)
DC2_DTLZ3	1.3824e-2 (1.30e-3) −	4.5198e-1 (1.99e-1) −	1.9546e-1 (2.54e-1) −	3.3823e-1 (2.97e-1) ≈	2.4488e-1 (2.60e-1) −	NaN (NaN)	4.0725e-1 (2.05e-1) −	5.5451e-1 (3.68e-3)
DC3_DTLZ1	4.6968e-1 (1.49e-1) −	5.2111e-1 (2.85e-3) −	5.2955e-2 (1.42e-1) −	5.2074e-1 (3.25e-3) −	2.3702e-1 (2.06e-1) −	1.5169e-2 (5.56e-2) −	5.3229e-1 (3.00e-3) ≈	5.3349e-1 (1.36e-3)
DC3_DTLZ3	0.0000e+0 (0.00e+0) −	3.5792e-1 (1.56e-2) ≈	4.2454e-2 (1.08e-1) −	1.1401e-2 (6.24e-2) −	4.0734e-2 (9.20e-2) −	0.0000e+0 (0.00e+0) −	1.7514e-1 (1.62e-1) −	3.5426e-1 (2.08e-2)
+/−/≈	2/3/1	0/5/1	0/6/0	0/5/1	0/6/0	0/4/0	0/5/1	

**Table 3:** Mean and standard deviation of IGD values on FCP; ’NAN’ indicates that no feasible solution was found; best result is highlighted in yellow.

Problem	BiCo	CTAEA	CAEAD	ICMA	PPS	ToP	TriP	CMOCPA
FCP1	NaN (NaN) −	NaN (NaN) −	NaN (NaN) −	3.5398e-2 (6.02e-4) −	NaN (NaN) −	NaN (NaN) −	NaN (NaN) −	3.2784e-2 (4.59e-4)
FCP2	NaN (NaN) −	NaN (NaN) −	NaN (NaN) −	3.0284e-2 (3.96e-3) −	NaN (NaN) −	NaN (NaN) −	NaN (NaN) −	2.6771e-2 (4.28e-4)
FCP3	NaN (NaN) −	NaN (NaN) −	NaN (NaN) −	3.8765e-2 (7.00e-4) −	NaN (NaN) −	NaN (NaN) −	NaN (NaN) −	3.5363e-2 (4.54e-4)
FCP4	NaN (NaN) −	NaN (NaN) −	NaN (NaN) −	2.7470e-2 (5.64e-4) −	NaN (NaN) −	NaN (NaN) −	NaN (NaN) −	2.5518e-2 (4.38e-4)
FCP5	4.6504e+0 (1.03e-1) −	4.7204e+0 (2.60e-2) −	4.6766e+0 (1.38e-3) −	1.7074e-1 (8.53e-1) −	4.6799e+0 (9.06e-3) −	4.2654e+0 (4.62e-3) −	4.6780e+0 (2.81e-3) −	1.3151e-2 (4.78e-4)
+/−/≈	0/5/0	0/5/0	0/5/0	0/5/0	0/5/0	0/5/0	0/5/0	

**Table 4:** Mean and standard deviation of HV values on FCP; ’NAN’ indicates that no feasible solution was found; best result is highlighted in yellow.

Problem	BiCo	CTAEA	CAEAD	ICMA	PPS	ToP	TriP	CMOCPA
FCP1	NaN (NaN) −	NaN (NaN) −	NaN (NaN) −	5.8138e-1 (1.19e-4) −	NaN (NaN) −	NaN (NaN) −	NaN (NaN) −	5.8168e-1 (1.12e-4)
FCP2	NaN (NaN) −	NaN (NaN) −	NaN (NaN) −	4.3132e-1 (2.76e-4) −	NaN (NaN) −	NaN (NaN −)	NaN (NaN) −	4.3161e-1 (7.19e-5)
FCP3	NaN (NaN) −	NaN (NaN) −	NaN (NaN) −	3.4691e-1 (1.15e-4) −	NaN (NaN) −	NaN (NaN) −	NaN (NaN) −	3.4706e-1 (1.14e-4)
FCP4	NaN (NaN) −	NaN (NaN) −	NaN (NaN) −	6.3405e-1 (2.62e-4) −	NaN (NaN) −	NaN (NaN) −	NaN (NaN) −	6.3455e-1 (6.25e-5)
FCP5	2.4822e-1 (5.28e-2) −	2.3073e-1 (2.28e-2) −	2.6215e-1 (3.70e-5) −	4.6845e-1 (3.95e-2) −	2.6120e-1 (2.35e-3) −	5.3823e-2 (2.44e-5) −	2.6209e-1 (2.20e-4) −	4.7986e-1 (1.03e-4)
+/−/≈	0/5/0	0/5/0	0/5/0	0/5/0	0/5/0	0/5/0	0/5/0	

**Table 5:** Mean and standard deviation of IGD values on DASCMOP; ’NAN’ indicates that no feasible solution was found; best result is highlighted in yellow.

Problem	BiCo	CTAEA	CAEAD	ICMA	PPS	ToP	TriP	CMOCPA
DASCMOP1	7.0996e-1 (3.86e-2) −	1.8326e-1 (1.41e-2) −	2.8895e-3 (2.16e-4) ≈	2.8510e-3 (2.42e-4) ≈	1.8950e-1 (2.31e-1) −	7.3320e-1 (1.51e-1) −	2.2514e-1 (2.21e-1) −	2.8431e-3 (1.31e-4)
DASCMOP2	2.3555e-1 (1.97e-2) −	9.1724e-2 (4.30e-2) −	4.1915e-3 (1.13e-4) +	4.1524e-3 (8.69e-5) +	5.1338e-3 (1.76e-4) −	4.8869e-1 (2.54e-1) −	4.4553e-3 (1.19e-4) ≈	4.4568e-3 (1.23e-4)
DASCMOP3	2.7163e-1 (3.32e-2) −	1.2803e-1 (1.04e-2) −	1.9162e-2 (1.27e-3) −	1.8978e-2 (2.15e-3) ≈	2.9289e-1 (1.07e-1) −	7.0016e-1 (1.08e-1) −	2.3729e-1 (1.23e-1) −	1.9088e-2 (1.76e-3)
DASCMOP4	1.2904e-3 (2.01e-4) −	1.0966e-2 (2.02e-3) −	1.8708e-3 (7.96e-4) −	1.7461e-3 (8.35e-4) −	1.5584e-1 (1.16e-1) −	NaN (NaN) −	1.6389e-3 (3.86e-4) −	1.2186e-3 (1.76e-4)
DASCMOP5	2.8730e-3 (7.70e-4) −	7.1383e-3 (4.82e-4) −	8.8972e-2 (2.26e-1) −	2.8957e-3 (8.51e-5) −	4.1381e-3 (3.64e-4) −	NaN (NaN) −	3.4980e-3 (8.80e-4) −	2.7905e-3 (5.03e-5)
DASCMOP6	4.7748e-2 (9.48e-2) −	2.3661e-2 (4.96e-3) −	8.0642e-2 (1.75e-1) ≈	1.9401e-2 (1.27e-3) −	1.6117e-1 (2.66e-1) −	NaN (NaN) −	1.8982e-2 (2.18e-3) −	1.8467e-2 (2.46e-3)
DASCMOP7	3.1651e-2 (8.35e-4) −	3.8141e-2 (7.98e-4) −	4.0516e-2 (1.78e-2) −	3.3291e-2 (1.05e-3) −	5.8472e-2 (1.20e-2) −	NaN (NaN) −	4.3108e-2 (3.44e-3) −	3.0844e-2 (7.42e-4)
DASCMOP8	4.1198e-2 (8.64e-4) −	5.7532e-2 (9.49e-3) −	7.1127e-2 (1.18e-1) −	4.3247e-2 (9.58e-4) −	6.9460e-2 (7.19e-3) −	NaN (NaN) −	5.4660e-2 (5.18e-3) −	3.9807e-2 (1.28e-3)
DASCMOP9	3.0573e-1 (5.44e-2) +	2.2504e-1 (7.20e-2) +	4.1371e-2 (1.06e-3) +	4.2363e-2 (5.90e-4) +	1.4368e-1 (1.10e-1) +	5.9957e-1 (2.05e-1) −	1.3114e-1 (7.30e-2) +	3.9880e-1 (1.07e-2)
+/−/≈	1/8/0	1/8/0	2/5/2	2/5/2	1/8/0	0/9/0	1/7/1	

**Table 6:** Mean and standard deviation of HV values on DASCMOP; ’NAN’ indicates that no feasible solution was found; best result is highlighted in yellow.

Problem	BiCo	CTAEA	CAEAD	ICMA	PPS	ToP	TriP	CMOCPA
DASCMOP1	1.0656e-2 (7.27e-3) −	1.6840e-1 (3.89e-3) −	2.1253e-1 (3.09e-4) −	2.1249e-1 (2.51e-4) −	1.7009e-1 (4.78e-2) −	1.2917e-2 (3.86e-2) −	1.6002e-1 (4.66e-2) −	2.1278e-1 (3.34e-4)
DASCMOP2	2.5508e-1 (3.92e-3) −	3.0983e-1 (1.26e-2) −	3.5530e-1 (9.54e-5) +	3.5554e-1 (4.64e-5) +	3.5483e-1 (9.92e-5) −	1.5024e-1 (1.11e-1) −	3.5514e-1 (7.22e-5) −	3.5519e-1 (8.41e-5)
DASCMOP3	2.1854e-1 (1.15e-2) −	2.6212e-1 (4.00e-3) −	3.1239e-1 (7.32e-5) +	3.1228e-1 (1.98e-4) ≈	2.2503e-1 (3.39e-2) −	3.5027e-2 (4.83e-2) −	2.3708e-1 (4.05e-2) −	3.1227e-1 (7.73e-5)
DASCMOP4	2.0413e-1 (3.72e-4) −	1.9675e-1 (4.02e-3) −	2.0349e-1 (3.80e-4) −	2.0378e-1 (2.07e-4) −	1.7059e-1 (2.52e-2) −	NaN (NaN) −	2.0398e-1 (2.36e-4) −	2.0424e-1 (1.15e-4)
DASCMOP5	3.5155e-1 (7.62e-4) +	3.4841e-1 (3.47e-4) −	3.1045e-1 (1.04e-1) −	3.5117e-1 (9.21e-5) −	3.5111e-1 (2.16e-4) −	NaN (NaN) −	3.5092e-1 (5.00e-4) −	3.5154e-1 (8.92e-5)
DASCMOP6	2.9357e-1 (5.30e-2) −	3.0889e-1 (3.32e-3) −	2.8328e-1 (8.17e-2) −	3.1236e-1 (3.22e-4) ≈	2.5075e-1 (1.14e-1) −	NaN (NaN) −	3.1222e-1 (3.18e-4) −	3.1244e-1 (1.03e-4)
DASCMOP7	2.8785e-1 (4.88e-4) ≈	2.8779e-1 (1.74e-4) −	2.8060e-1 (1.03e-2) −	2.8782e-1 (3.85e-4) ≈	2.7840e-1 (6.51e-3) −	NaN (NaN) −	2.8478e-1 (7.51e-4) −	2.8796e-1 (2.79e-4)
DASCMOP8	2.0671e-1 (5.31e-4) ≈	2.0321e-1 (2.04e-3) −	1.9222e-1 (3.95e-2) −	2.0747e-1 (2.50e-4) +	2.0051e-1 (1.65e-3) −	NaN (NaN) −	2.0400e-1 (8.52e-4) −	2.0654e-1 (3.66e-4)
DASCMOP9	1.3765e-1 (9.72e-3) +	1.5234e-1 (1.55e-2) +	2.0490e-1 (4.52e-4) +	2.0750e-1 (1.82e-4) +	1.7760e-1 (2.63e-2) +	8.6488e-2 (3.73e-2) −	1.7815e-1 (2.13e-2) +	1.2667e-1 (3.47e-3)
+/−/≈	2/5/2	1/8/0	3/6/0	3/3/3	1/8/0	0/9/0	1/8/0	

**Table 7:** Mean and standard deviation of IGD values on ZDT and DTLZ; ’NAN’ indicates that no feasible solution was found; best result is highlighted in yellow.

Problem	MOEAD	eMOEA	MOPSO	NSGAII	SPEA2	KnEA	GrEA	CMOCPA
ZDT1	5.0328e-3 (1.04e-3) −	2.8606e-2 (1.82e-3) −	1.6802e+0 (9.47e-2) −	4.7933e-3 (2.19e-4) −	3.9617e-3 (7.27e-5) −	1.6000e-1 (8.99e-2) −	7.7519e-3 (2.03e-3) −	3.8891e-3 (7.68e-5)
ZDT2	5.8825e-3 (9.54e-4) −	3.0931e-2 (3.38e-3) −	3.1778e+0 (1.64e-1) −	4.8971e-3 (2.04e-4) −	3.9216e-3 (4.55e-5) −	9.4728e-2 (2.16e-2) −	7.9482e-3 (1.44e-4) −	3.8472e-3 (4.65e-5)
ZDT3	1.4930e-2 (6.44e-3) −	6.6691e-2 (1.16e-2) −	1.1959e+0 (7.17e-2) −	6.3819e-3 (5.33e-3) −	4.9250e-3 (1.08e-4) −	1.0725e-2 (5.43e-3) −	1.3962e-2 (9.57e-4) −	4.8095e-3 (8.46e-5)
ZDT4	7.4937e-3 (2.13e-3) −	2.8669e-2 (1.79e-3) −	1.3118e+1 (5.26e+0) −	4.8101e-3 (4.12e-4) −	4.0406e-3 (2.35e-4) +	2.5345e-1 (9.47e-2) −	3.3221e-1 (1.42e-1) −	4.2835e-3 (3.62e-4)
ZDT6	4.6155e-3 (5.88e-4) −	2.9127e-2 (1.43e-3) −	5.5098e+0 (4.97e-1) −	3.6581e-3 (9.10e-5) −	3.0849e-3 (2.45e-5) −	7.2564e-3 (1.77e-3) −	6.0337e-3 (9.05e-5) −	3.0663e-3 (3.14e-5)
DTLZ1	2.0639e-2 (7.92e-5) −	3.6655e-2 (2.28e-3) −	1.5378e+0 (1.01e+0) −	2.6598e-2 (1.03e-3) −	2.0254e-2 (2.38e-4) ≈	5.2936e-2 (2.87e-2) −	9.0384e-2 (7.49e-2) −	2.0306e-2 (2.48e-4)
DTLZ2	5.4464e-2 (4.51e-7) −	6.4801e-2 (1.33e-3) −	1.5407e-1 (3.38e-2) −	6.9652e-2 (2.37e-3) −	5.4266e-2 (5.08e-4) −	6.6606e-2 (3.09e-3) −	6.3790e-2 (5.49e-4) −	5.3337e-2 (3.36e-4)
DTLZ3	5.9931e-2 (5.59e-3) +	1.3317e-1 (1.99e-1) ≈	1.4438e+1 (6.98e+0) −	7.1776e-2 (4.07e-3) ≈	5.6580e-2 (2.43e-3) +	9.6273e-2 (2.15e-2) +	1.7125e-1 (1.41e-1) ≈	2.9649e-1 (4.96e-1)
DTLZ4	2.7931e-1 (2.71e-1) −	2.5799e-1 (2.65e-1) −	2.6746e-1 (1.92e-1) −	9.6517e-2 (1.60e-1) +	2.7599e-1 (2.93e-1) −	1.2482e-1 (2.23e-1) −	1.3005e-1 (1.65e-1) −	1.1868e-1 (1.68e-1)
DTLZ5	3.3860e-2 (2.79e-5) −	6.8262e-2 (4.12e-3) −	9.0944e-3 (1.13e-3) −	5.8083e-3 (2.78e-4) −	4.4163e-3 (1.16e-4) −	9.2901e-3 (1.27e-3) −	2.1806e-2 (1.11e-3) −	4.2786e-3 (1.17e-4)
DTLZ6	3.3911e-2 (1.26e-5) −	6.2350e-2 (2.03e-3) −	9.2118e+0 (6.70e-2) −	5.8309e-3 (3.81e-4) −	4.0878e-3 (3.84e-5) −	1.2456e-2 (6.70e-3) −	2.2303e-2 (9.19e-5) −	4.0298e-3 (2.55e-5)
DTLZ7	1.9844e-1 (1.64e-1) −	2.2912e-1 (1.90e-1) −	6.5150e+0 (8.39e-1) −	7.6159e-2 (4.40e-3) −	6.9672e-2 (5.21e-2) ≈	7.4722e-2 (5.24e-2) −	9.2857e-2 (5.53e-2) −	5.9811e-2 (1.27e-3)
+/−/≈	1/11/0	0/11/1	0/12/0	1/10/1	2/8/2	1/11/0	0/11/1	

**Table 8:** Mean and standard deviation of HV values on ZDT and DTLZ; ’NAN’ indicates that no feasible solution was found; best result is highlighted in yellow.

Problem	MOEAD	eMOEA	MOPSO	NSGAII	SPEA2	KnEA	GrEA	CMOCPA
ZDT1	7.1781e-1 (1.33e-3) −	6.8614e-1 (3.32e-3) −	0.0000e+0 (0.00e+0) −	7.1920e-1 (2.73e-4) −	7.2028e-1 (1.27e-4) −	6.2622e-1 (5.17e-2) −	7.1514e-1 (2.02e-3) −	7.2035e-1 (1.18e-4)
ZDT2	4.4031e-1 (2.06e-3) −	4.1083e-1 (3.17e-3) −	0.0000e+0 (0.00e+0) −	4.4398e-1 (2.26e-4) −	4.4497e-1 (8.60e-5) ≈	3.5749e-1 (1.87e-2) −	4.4153e-1 (5.66e-5) −	4.4500e-1 (9.56e-5)
ZDT3	6.0068e-1 (1.76e-2) ≈	5.8614e-1 (2.92e-2) −	4.4309e-4 (1.19e-3) −	6.0236e-1 (1.62e-2) +	5.9960e-1 (5.83e-5) −	6.0128e-1 (1.62e-2) +	5.9728e-1 (3.68e-4) −	5.9964e-1 (6.12e-5)
ZDT4	7.1281e-1 (3.03e-3) −	6.8414e-1 (4.70e-3) −	0.0000e+0 (0.00e+0) −	7.1866e-1 (1.03e-3) ≈	7.1956e-1 (7.55e-4) +	5.6968e-1 (5.82e-2) −	5.1348e-1 (9.92e-2) −	7.1893e-1 (8.80e-4)
ZDT6	3.8551e-1 (1.00e-3) −	3.5878e-1 (2.06e-3) −	0.0000e+0 (0.00e+0) −	3.8830e-1 (1.06e-4) −	3.8888e-1 (4.50e-5) +	3.8476e-1 (1.74e-3) −	3.8599e-1 (9.16e-5) −	3.8876e-1 (1.32e-4)
DTLZ1	8.4079e-1 (7.47e-4) ≈	7.2735e-1 (1.70e-2) −	3.3631e-4 (1.84e-3) −	8.2427e-1 (3.39e-3) −	8.4138e-1 (1.40e-3) +	7.4896e-1 (5.46e-2) −	6.7294e-1 (1.42e-1) −	8.4024e-1 (1.43e-3)
DTLZ2	5.5961e-1 (5.00e-6) +	5.4650e-1 (2.54e-3) −	4.1184e-1 (2.92e-2) −	5.3141e-1 (4.33e-3) −	5.5504e-1 (8.65e-4) −	5.4381e-1 (3.60e-3) −	5.5845e-1 (5.77e-4) +	5.5764e-1 (1.22e-3)
DTLZ3	5.3562e-1 (1.76e-2) +	4.7263e-1 (1.20e-1) ≈	0.0000e+0 (0.00e+0) −	5.1877e-1 (1.37e-2) ≈	5.4425e-1 (7.96e-3) +	4.9667e-1 (2.72e-2) ≈	4.5856e-1 (1.10e-1) ≈	4.0698e-1 (2.08e-1)
DTLZ4	4.5608e-1 (1.27e-1) ≈	4.5492e-1 (1.34e-1) −	3.1868e-1 (7.29e-2) −	5.2074e-1 (8.13e-2) −	4.5444e-1 (1.39e-1) −	5.1446e-1 (1.15e-1) −	5.2844e-1 (7.88e-2) −	5.2970e-1 (7.28e-2)
DTLZ5	1.8188e-1 (1.51e-5) −	1.6809e-1 (1.83e-3) −	1.9392e-1 (2.90e-3) −	1.9912e-1 (1.91e-4) −	1.9954e-1 (1.49e-4) −	1.9408e-1 (1.39e-3) −	1.8813e-1 (4.94e-4) −	1.9977e-1 (1.13e-4)
DTLZ6	1.8185e-1 (6.54e-6) −	1.7722e-1 (6.68e-4) −	0.0000e+0 (0.00e+0) −	1.9945e-1 (1.53e-4) −	2.0006e-1 (4.95e-5) −	1.9229e-1 (5.94e-3) −	1.8765e-1 (2.83e-5) −	2.0010e-1 (3.57e-5)
DTLZ7	2.5236e-1 (1.36e-2) −	2.4692e-1 (2.12e-2) −	0.0000e+0 (0.00e+0) −	2.6839e-1 (1.90e-3) −	2.7575e-1 (6.22e-3) −	2.7673e-1 (7.07e-3) −	2.7036e-1 (7.10e-3) −	2.7737e-1 (7.87e-4)
+/−/≈	2/7/3	0/11/1	0/12/0	1/9/2	4/7/1	1/10/1	1/10/1	

**Table 9:** Mean and standard deviation of IGD values on mechanical design problems of RWMOPs; ’NAN’ indicates that no feasible solution was found; best result is highlighted in yellow.

Problem	MOEAD	eMOEA	MOPSO	NSGAII	SPEA2	KnEA	GrEA	CMOCPA
RWMOP1	NaN (NaN)−	NaN (NaN)−	NaN (NaN)−	3.5961e+5 (8.69e+1) ≈	NaN (NaN)−	4.8572e+5 (2.15e+5) −	NaN (NaN)−	3.5990e+5 (8.16e+2)
RWMOP2	NaN (NaN)−	NaN (NaN)−	NaN (NaN)−	5.4112e+1 (3.17e+1) ≈	NaN (NaN)−	5.6426e+1 (3.13e+1) ≈	NaN (NaN)−	6.1584e+1 (2.91e+1)
RWMOP3	NaN (NaN)−	3.7831e+4 (0.00e+0) ≈	9.9252e+14 (5.36e+15) −	2.2125e+2 (3.47e+2) +	NaN (NaN)−	1.4399e+4 (5.80e+3) −	NaN (NaN)−	1.2378e+3 (1.15e+3)
RWMOP4	NaN (NaN)−	NaN (NaN)−	2.4187e+4 (6.09e+4) −	1.2702e+0 (1.01e-1) +	NaN (NaN)−	7.4940e+0 (2.95e+0) −	NaN (NaN)−	1.3552e+0 (1.36e-2)
RWMOP5	NaN (NaN)−	1.8879e+0 (3.85e-3) +	NaN (NaN)−	1.8882e+0 (4.11e-4) −	1.8882e+0 (5.89e-5) −	2.7303e+0 (3.24e-1) −	1.8884e+0 (2.38e-4) −	1.8881e+0 (1.31e-4)
RWMOP6	NaN (NaN)−	NaN (NaN)−	NaN (NaN)−	6.9699e+2 (3.01e+2) −	NaN (NaN)	2.2970e+3 (8.40e+2) −	NaN (NaN)−	6.0571e+2 (1.00e+0)
RWMOP7	1.5430e+1 (1.36e-1) −	1.4185e+1 (1.37e+0) ≈	2.4624e+1 (1.15e+1) −	1.3810e+1 (3.03e+0) ≈	1.4086e+1 (2.87e+0) ≈	1.5318e+1 (3.89e-1) −	1.4800e+1 (6.98e-1) −	1.2785e+1 (3.59e+0)
RWMOP8	NaN (NaN)−	2.6709e+1 (1.61e+1) −	1.2085e+1 (1.26e+1) −	2.1298e+0 (1.89e-4) −	2.1235e+0 (3.50e-2) ≈	1.6635e+1 (1.45e+1) −	6.0661e+0 (4.50e+0) −	2.1098e+0 (3.05e-2)
RWMOP9	1.6484e+3 (3.49e-2) −	3.8763e-2 (6.37e-3) −	9.9729e+13 (3.62e+14) −	3.7239e-2 (1.41e-17) ≈	3.7239e-2 (1.98e-12) ≈	5.8747e+2 (1.22e+2) −	2.5868e+2 (8.81e+1) −	3.7239e-2 (1.41e-17)
RWMOP10	1.5861e+2 (9.63e-5) −	1.6677e-3 (2.04e-3) ≈	1.9116e+44 (1.03e+45) −	7.5315e-3 (7.66e-3) ≈	5.9892e-3 (4.16e-3) ≈	8.2014e+0 (3.77e+0) −	1.2891e+2 (1.55e+1) −	5.5261e-3 (7.46e-3)
RWMOP11	3.7069e+6 (8.20e+2) −	2.5043e+6 (9.02e+3) −	1.1797e+7 (1.38e+7) −	2.4589e+6 (4.09e+4) −	2.4906e+6 (2.35e+5) −	2.4559e+6 (5.11e+4) −	2.5608e+6 (3.70e+4) −	2.3088e+6 (7.19e+4)
RWMOP12	NaN (NaN)−	4.5903e+1 (0.00e+0) ≈	8.7901e+2 (9.82e+2) −	3.7516e+0 (2.45e+0) −	2.5646e+0 (1.64e+0) ≈	1.3507e+0 (7.96e-1) +	NaN (NaN)−	2.0109e+0 (1.12e+0)
RWMOP13	NaN (NaN)−	NaN (NaN)−	NaN (NaN)−	4.5851e+2 (6.80e+1) −	NaN (NaN)−	8.0857e+2 (9.97e+1) −	NaN (NaN)−	3.8510e+2 (1.99e+1)
RWMOP14	NaN (NaN)−	1.2264e-2 (5.87e-4) −	1.8289e+3 (0.00e+0) −	1.2137e-2 (2.12e-14) ≈	1.2137e-2 (2.35e-12) ≈	6.5289e-1 (5.18e-2) −	NaN (NaN)−	1.2137e-2 (3.53e-18)
RWMOP15	NaN (NaN)−	NaN (NaN)−	NaN (NaN)−	4.3236e+3 (4.14e+3) −	NaN (NaN)−	2.9551e+4 (1.67e+4) −	NaN (NaN)−	5.7265e+2 (8.93e+2)
RWMOP16	NaN (NaN)−	1.9989e-3 (1.32e-18) ≈	2.0269e+6 (7.98e+6) −	1.9989e-3 (1.32e-18) ≈	1.9989e-3 (1.32e-18) ≈	5.9503e-2 (4.32e-2) −	2.4842e+0 (1.13e-1) −	1.9989e-3 (1.32e-18)
RWMOP17	4.4783e+9 (3.75e+9) −	NaN (NaN)−	8.1597e+3 (5.52e+2) −	5.1163e+3 (2.79e+2) −	NaN (NaN)−	1.1209e+4 (1.84e+3) −	NaN (NaN)−	4.6960e+3 (1.53e+2)
RWMOP18	9.4571e-2 (3.59e-6) −	1.0726e-1 (1.89e-2) −	NaN (NaN)−	9.4283e-2 (2.02e-4) +	9.4458e-2 (1.25e-4) ≈	9.4784e-2 (8.18e-4) ≈	9.4414e-2 (2.61e-4) ≈	9.4514e-2 (8.82e-5)
RWMOP19	NaN (NaN)−	NaN (NaN)−	NaN (NaN)−	7.7965e+4 (2.50e+4) −	NaN (NaN)−	1.3088e+5 (8.95e+3) −	NaN (NaN)−	3.8623e+4 (1.01e+4)
RWMOP20	NaN (NaN)−	NaN (NaN)−	NaN (NaN)−	1.9764e+3 (2.05e+2) +	NaN (NaN)−	1.9680e+3 (2.24e+2) +	NaN (NaN)−	2.0864e+3 (1.73e+2)
RWMOP21	6.6237e+0 (3.95e-3) −	1.4326e-1 (2.92e-1) −	4.9022e+0 (6.43e+0) −	1.3186e-1 (2.36e-1) −	7.0673e-2 (1.25e-1) ≈	2.4790e+0 (3.75e-1) −	4.4463e-1 (3.76e-1) −	1.6050e-2 (7.06e-18)
+/−/≈	0/21/0	1/15/5	0/21/0	4/10/7	0/12/9	2/17/2	0/20/1	

**Table 10:** Mean and standard deviation of HV values on mechanical design problems of RWMOPs; ’NAN’ indicates that no feasible solution was found; best result is highlighted in yellow.

Problem	MOEAD	eMOEA	MOPSO	NSGAII	SPEA2	KnEA	GrEA	CMOCPA
RWMOP1	NaN (NaN)−	NaN (NaN)−	NaN (NaN)−	6.0523e-1 (4.27e-4) −	NaN (NaN)−	5.9323e-1 (1.41e-2) −	NaN (NaN)−	6.0766e-1 (3.43e-4)
RWMOP2	NaN (NaN)−	NaN (NaN)−	NaN (NaN)−	2.2625e-1 (1.49e-1) ≈	NaN (NaN)−	2.3799e-1 (1.43e-1) ≈	NaN (NaN)−	2.4536e-1 (1.52e-1)
RWMOP3	NaN (NaN)−	4.0552e-1 (0.00e+0) ≈	8.9848e-1 (2.44e-1) −	9.0206e-1 (1.68e-4) +	NaN (NaN)−	8.3815e-1 (4.08e-2) −	NaN (NaN)−	9.0010e-1 (5.88e-4)
RWMOP4	NaN (NaN)−	NaN (NaN)−	8.8056e-1 (3.06e-1) +	8.5888e-1 (3.41e-3) +	NaN (NaN)−	7.6779e-1 (2.96e-2) −	NaN (NaN)−	8.5619e-1 (1.49e-3)
RWMOP5	NaN (NaN−)	2.4936e-1 (1.38e-3) −	NaN (NaN)−	4.3356e-1 (1.14e-3) ≈	2.7321e-1 (2.58e-3) −	3.9767e-1 (1.27e-2) −	2.7424e-1 (1.05e-3) −	4.3417e-1 (1.80e-4)
RWMOP6	NaN (NaN)−	NaN (NaN)−	NaN (NaN)−	2.7715e-1 (5.39e-5) +	NaN (NaN)−	2.4092e-1 (3.09e-2) −	NaN (NaN)−	2.7482e-1 (1.07e-3)
RWMOP7	4.7784e-1 (3.64e-3) −	4.8285e-1 (6.94e-4) −	7.5277e-1 (1.72e-1) +	4.8398e-1 (6.83e-5) −	4.8285e-1 (1.92e-4) −	4.8218e-1 (9.93e-4) −	4.8178e-1 (4.08e-4) −	4.8442e-1 (7.98e-5)
RWMOP8	NaN (NaN)−	2.1196e-2 (2.09e-3) −	4.0212e-2 (1.46e-2) +	2.5879e-2 (1.04e-4) ≈	2.3654e-2 (4.28e-4) −	2.5050e-2 (5.47e-4) −	2.2567e-2 (3.27e-4) −	2.5794e-2 (1.85e-4)
RWMOP9	5.3068e-2 (5.05e-5) −	2.5125e-1 (5.21e-2) −	6.1315e-1 (1.80e-1) +	4.0902e-1 (1.49e-4) −	4.0947e-1 (1.13e-4) ≈	3.6925e-1 (8.76e-3) −	4.0115e-1 (2.42e-3) −	4.0950e-1 (1.19e-4)
RWMOP10	7.9369e-2 (6.24e-4) −	5.7450e-1 (2.29e-1) −	6.5537e-1 (2.93e-1) ≈	8.4728e-1 (2.23e-4) +	8.4208e-1 (1.28e-3) ≈	8.2504e-1 (1.10e-2) −	8.3621e-1 (5.73e-3) −	8.4218e-1 (1.23e-3)
RWMOP11	5.7358e-2 (9.23e-4) −	1.0791e-1 (1.96e-4) +	6.7100e-3 (1.43e-2) −	9.4453e-2 (1.33e-3) +	6.1777e-2 (9.98e-3) −	9.7678e-2 (1.22e-3) +	8.4401e-2 (4.47e-3) −	9.2711e-2 (2.09e-3)
RWMOP12	NaN (NaN)−	0.0000e+0 (0.00e+0) ≈	5.9992e-1 (2.70e-1) ≈	5.5982e-1 (3.88e-4) +	5.3842e-1 (7.14e-3) −	5.2949e-1 (6.15e-3) −	NaN (NaN)	5.5653e-1 (1.25e-3)
RWMOP13	NaN (NaN)−	NaN (NaN)−	NaN (NaN)−	8.7936e-2 (1.04e-4) +	NaN (NaN)−	8.7546e-2 (3.07e-4) ≈	NaN (NaN)−	8.7523e-2 (1.18e-4)
RWMOP14	NaN (NaN)−	1.4558e-1 (7.25e-2) −	9.9956e-1 (0.00e+0) ≈	6.1748e-1 (1.31e-3) +	3.4771e-1 (2.64e-3) −	5.9485e-1 (9.49e-3) −	NaN (NaN)−	6.1463e-1 (7.00e-4)
RWMOP15	NaN (NaN)−	NaN (NaN)−	NaN (NaN)−	5.4143e-1 (1.33e-3) −	NaN (NaN)−	4.8584e-1 (2.84e-2) −	NaN (NaN)−	5.4226e-1 (1.81e-4)
RWMOP16	NaN (NaN)−	3.4033e-1 (1.67e-1) −	3.3888e-1 (3.36e-1) −	7.6373e-1 (1.45e-4) +	7.6167e-1 (3.80e-4) −	7.6174e-1 (1.31e-3) −	3.5934e-1 (1.44e-1) −	7.6251e-1 (1.86e-4)
RWMOP17	2.0615e-1 (1.35e-1) ≈	NaN (NaN)−	6.1754e-1 (2.31e-2) +	2.6369e-1 (9.01e-3) −	NaN (NaN)−	4.3461e-1 (1.12e+0) +	NaN (NaN)−	2.6714e-1 (9.74e-3)
RWMOP18	4.0246e-2 (3.71e-5) −	2.9443e-2 (2.75e-3) −	NaN (NaN)−	4.0493e-2 (4.44e-6) −	4.0401e-2 (5.19e-5) −	3.8136e-2 (7.82e-4) −	4.0234e-2 (9.59e-5) −	4.0509e-2 (4.24e-6)
RWMOP19	NaN (NaN)−	NaN (NaN)−	NaN (NaN)−	3.3547e-1 (9.62e-3) −	NaN (NaN)−	2.8196e-1 (1.97e-2) −	NaN (NaN)−	3.6157e-1 (2.88e-3)
RWMOP20	NaN (NaN)−	NaN (NaN)−	NaN (NaN)−	0.0000e+0 (0.00e+0) ≈	NaN (NaN)−	0.0000e+0 (0.00e+0) ≈	NaN (NaN)−	0.0000e+0 (0.00e+0)
RWMOP21	2.9319e-2 (3.47e-6) −	3.0724e-2 (6.57e-4) −	6.6135e-2 (1.59e-2) +	3.1741e-2 (2.10e-5) −	3.1721e-2 (1.59e-4) −	2.5192e-2 (8.29e-4) −	3.1485e-2 (3.69e-4) −	3.1761e-2 (7.50e-7)
+/−/≈	0/20/1	1/18/2	6/12/3	9/8/4	0/19/2	2/16/3	0/21/0	

**Table 11:** Mean and standard deviation of IGD values on chemical engineering problems of RWMOPs; ’NAN’ indicates that no feasible solution was found; best result is highlighted in yellow.

Problem	MOEAD	eMOEA	MOPSO	NSGAII	SPEA2	KnEA	GrEA	CMOCPA
RWMOP22	NaN (NaN)−	NaN (NaN)−	NaN (NaN)−	NaN (NaN)−	NaN (NaN)−	NaN (NaN)−	NaN (NaN)−	1.7024e+3 (3.64e+2)
RWMOP23	NaN (NaN)−	NaN (NaN)−	NaN (NaN)−	1.2492e+0 (7.02e-1) ≈	NaN (NaN)−	1.0692e+0 (5.86e-1) ≈	NaN (NaN)−	8.8150e-1 (4.90e-1)
RWMOP24	NaN (NaN)−	NaN (NaN)−	NaN (NaN)−	NaN (NaN)−	NaN (NaN)−	NaN (NaN)−	NaN (NaN)−	1.7997e+4 (6.57e+4)
+/−/≈	0/3/0	0/3/0	0/3/0	0/2/1	0/3/0	0/2/1	0/3/0	

**Table 12:** Mean and standard deviation of HV values on chemical engineering problems of RWMOPs; ’NAN’ indicates that no feasible solution was found; best result is highlighted in yellow.

Problem	MOEAD	eMOEA	MOPSO	NSGAII	SPEA2	KnEA	GrEA	CMOCPA
RWMOP22	NaN (NaN)−	NaN (NaN)−	NaN (NaN)−	NaN (NaN)−	NaN (NaN)−	NaN (NaN)−	NaN (NaN)−	8.2709e-1 (2.16e-1)
RWMOP23	NaN (NaN)−	NaN (NaN)−	NaN (NaN)−	3.6105e-1 (1.72e-1) ≈	NaN (NaN)−	3.1904e-1 (1.43e-1) ≈	NaN (NaN)−	2.6831e-1 (1.29e-1)
RWMOP24	NaN (NaN)−	NaN (NaN)−	NaN (NaN)−	NaN (NaN)	NaN (NaN)−	NaN (NaN)−	NaN (NaN)−	4.2171e-1 (4.37e-1)
+/−/≈	0/3/0	0/3/0	0/3/0	0/2/1	0/3/0	0/2/1	0/3/0	

**Table 13:** Mean and standard deviation of IGD values on process, design and synthesis problems of RWMOPs; ’NAN’ indicates that no feasible solution was found; best result is highlighted in yellow.

Problem	MOEAD	eMOEA	MOPSO	NSGAII	SPEA2	KnEA	GrEA	CMOCPA
RWMOP25	8.0323e-1 (1.17e-1) −	7.4475e-1 (5.58e-4) −	6.2586e+2 (1.19e+3) −	7.4391e-1 (9.77e-5) ≈	7.4408e-1 (2.09e-4) −	7.4388e-1 (3.03e-5) ≈	7.4389e-1 (7.73e-7) −	7.4388e-1 (1.90e-5)
RWMOP26	NaN (NaN)−	NaN (NaN)−	NaN (NaN)−	2.6894e-1 (2.97e-2) −	NaN (NaN)−	2.8814e-1 (4.48e-2) −	NaN (NaN)−	2.4734e-1 (1.45e-4)
RWMOP27	1.0629e+0 (7.93e-2) −	1.0306e+0 (7.22e-2) −	1.7992e+0 (9.59e-2) −	9.9000e-1 (1.90e-5) −	1.0138e+0 (4.90e-2) −	9.9000e-1 (5.11e-5) ≈	1.0452e+0 (3.53e-2) −	9.8997e-1 (1.36e-4)
RWMOP28	NaN (NaN)−	NaN (NaN)−	NaN (NaN)−	NaN (NaN)−	NaN (NaN)−	NaN (NaN)−	NaN (NaN)−	8.8494e+0 (0.00e+0)
RWMOP29	NaN (NaN)−	NaN (NaN)−	NaN (NaN)−	9.4792e+0 (6.64e-1) ≈	NaN (NaN)−	9.7243e+0 (9.16e-1) ≈	NaN (NaN)−	9.2095e+0 (1.07e-1)
+/−/≈	0/5/0	0/5/0	0/5/0	0/3/2	0/5/0	0/2/3	0/5/0	

**Table 14:** Mean and standard deviation of HV values on process, design and synthesis problems of RWMOPs; ’NAN’ indicates that no feasible solution was found; best result is highlighted in yellow.

Problem	MOEAD	eMOEA	MOPSO	NSGAII	SPEA2	KnEA	GrEA	CMOCPA
RWMOP25	2.6578e-1 (6.93e-2) +	2.2908e-1 (2.17e-3) −	9.9924e-1 (3.46e-3) +	2.4107e-1 (6.17e-5) −	2.3465e-1 (1.08e-3) −	2.4096e-1 (2.58e-4) −	2.3126e-1 (2.20e-5) −	2.4150e-1 (1.09e-5)
RWMOP26	NaN (NaN)−	NaN (NaN)−	NaN (NaN)−	1.4281e-1 (2.31e-2) ≈	NaN (NaN)−	1.3589e-1 (2.77e-2) −	NaN (NaN)−	1.4605e-1 (4.41e-3)
RWMOP27	4.3841e+1 (5.17e+1) −	2.6239e+10 (1.03e+11) ≈	6.6799e+0 (4.14e-1) −	1.8438e+10 (4.67e+10) +	3.8284e+9 (1.73e+10) +	2.1712e+11 (1.17e+12) +	3.3008e+1 (3.04e+1) −	1.2478e+9 (5.86e+9)
RWMOP28	NaN (NaN)−	NaN (NaN)−	NaN (NaN)−	NaN (NaN)−	NaN (NaN)−	NaN (NaN)−	NaN (NaN)−	1.6667e-2 (0.00e+0)
RWMOP29	NaN (NaN)−	NaN (NaN)−	NaN (NaN)−	7.5346e-1 (5.86e-2) ≈	NaN (NaN)−	7.4404e-1 (6.86e-2) −	NaN (NaN)−	7.8077e-1 (2.47e-3)
+/−/≈	1/4/0	0/4/1	1/4/0	1/2/2	1/4/0	1/4/0	0/5/0	

**Table 15:** Mean and standard deviation of IGD values on power electronics problems of RWMOPs; ’NAN’ indicates that no feasible solution was found; best result is highlighted in yellow.

Problem	MOEAD	eMOEA	MOPSO	NSGAII	SPEA2	KnEA	GrEA	CMOCPA
RWMOP30	NaN (NaN)−	NaN (NaN)−	NaN (NaN)−	1.2752e-1 (2.88e-2) ≈	NaN (NaN)−	1.6247e-1 (3.73e-2) −	NaN (NaN)−	1.1106e-1 (3.92e-2)
RWMOP31	NaN (NaN)−	NaN (NaN)−	NaN (NaN)−	1.7148e-1 (1.14e-1) ≈	NaN (NaN)−	1.9880e-1 (1.65e-1) ≈	NaN (NaN)−	1.5409e-1 (1.27e-1)
RWMOP32	NaN (NaN)−	NaN (NaN)−	NaN (NaN)−	2.6653e-1 (1.00e-1) ≈	NaN (NaN)−	3.6026e-1 (1.55e-1) −	NaN (NaN)−	1.6997e-1 (1.15e-1)
RWMOP33	NaN (NaN)−	NaN (NaN)−	NaN (NaN)−	2.1002e+0 (7.05e-1) ≈	NaN (NaN)−	2.1525e+0 (8.35e-1) ≈	NaN (NaN)−	3.0123e+0 (3.65e-1)
RWMOP34	NaN (NaN)−	NaN (NaN)−	NaN (NaN)−	1.6020e+0 (8.63e-1) ≈	NaN (NaN)−	1.5357e+0 (8.71e-1) ≈	NaN (NaN)−	3.0357e+0 (1.14e+0)
RWMOP35	NaN (NaN)−	NaN (NaN)−	NaN (NaN)−	4.2593e+0 (1.57e+0) ≈	NaN (NaN)−	4.7182e+0 (1.74e+0) −	NaN (NaN)−	2.7377e+0 (1.14e+0)
+/−/≈	0/6/0	0/6/0	0/6/0	0/0/6	0/6/0	0/3/3	0/6/0	

**Table 16:** Mean and standard deviation of HV values on power electronics problems of RWMOPs; ’NAN’ indicates that no feasible solution was found; best result is highlighted in yellow.

Problem	MOEAD	eMOEA	MOPSO	NSGAII	SPEA2	KnEA	GrEA	CMOCPA
RWMOP30	NaN (NaN)−	NaN (NaN)−	NaN (NaN)−	5.7608e-1 (1.18e-1) ≈	NaN (NaN)−	5.6748e-1 (1.05e-1) ≈	NaN (NaN)−	4.8258e-1 (2.16e-1)
RWMOP31	NaN (NaN)−	NaN (NaN)−	NaN (NaN)−	4.9581e-1 (2.99e-1) ≈	NaN (NaN)−	4.3421e-1 (2.71e-1) ≈	NaN (NaN)−	3.1568e-1 (3.25e-1)
RWMOP32	NaN (NaN)−	NaN (NaN)−	NaN (NaN)−	7.2496e-1 (1.06e-1) +	NaN (NaN)	6.6083e-1 (1.92e-1) +	NaN (NaN)	4.0098e-1 (3.12e-1)
RWMOP33	NaN (NaN)−	NaN (NaN)−	NaN (NaN)−	0.0000e+0 (0.00e+0) ≈	NaN (NaN)−	0.0000e+0 (0.00e+0) ≈	NaN (NaN)−	0.0000e+0 (0.00e+0)
RWMOP34	NaN (NaN)−	NaN (NaN)−	NaN (NaN)−	0.0000e+0 (0.00e+0) ≈	NaN (NaN)−	0.0000e+0 (0.00e+0) ≈	NaN (NaN)−	0.0000e+0 (0.00e+0)
RWMOP35	NaN (NaN)−	NaN (NaN)−	NaN (NaN)−	4.6596e-1 (1.49e-1) +	NaN (NaN)−	4.8053e-1 (1.21e-1) +	NaN (NaN)−	3.2464e-1 (1.62e-1)
+/−/≈	0/6/0	0/6/0	0/6/0	2/0/4	0/6/0	2/0/4	0/6/0	

**Table 17:** Mean and standard deviation of IGD values on power system optimization problems of RWMOPs; ’NAN’ indicates that no feasible solution was found; best result is highlighted in yellow.

Problem	MOEAD	eMOEA	MOPSO	NSGAII	SPEA2	KnEA	GrEA	CMOCPA
RWMOP36	NaN (NaN) ≈	NaN (NaN) ≈	NaN (NaN) ≈	NaN (NaN) ≈	NaN (NaN) ≈	NaN (NaN) ≈	NaN (NaN) ≈	NaN (NaN) ≈
RWMOP37	NaN (NaN) ≈	NaN (NaN) ≈	NaN (NaN) ≈	NaN (NaN) ≈	NaN (NaN) ≈	NaN (NaN) ≈	NaN (NaN) ≈	NaN (NaN) ≈
RWMOP38	NaN (NaN) ≈	NaN (NaN) ≈	NaN (NaN) ≈	NaN (NaN) ≈	NaN (NaN) ≈	NaN (NaN) ≈	NaN (NaN) ≈	NaN (NaN) ≈
RWMOP39	NaN (NaN) ≈	NaN (NaN) ≈	NaN (NaN) ≈	NaN (NaN) ≈	NaN (NaN) ≈	NaN (NaN) ≈	NaN (NaN) ≈	NaN (NaN) ≈
RWMOP40	NaN (NaN) ≈	NaN (NaN) ≈	NaN (NaN) ≈	NaN (NaN) ≈	NaN (NaN) ≈	NaN (NaN) ≈	NaN (NaN) ≈	NaN (NaN) ≈
RWMOP41	NaN (NaN) ≈	NaN (NaN) ≈	NaN (NaN) ≈	NaN (NaN) ≈	NaN (NaN) ≈	NaN (NaN) ≈	NaN (NaN) ≈	NaN (NaN) ≈
RWMOP42	NaN (NaN) ≈	NaN (NaN) ≈	NaN (NaN) ≈	NaN (NaN) ≈	NaN (NaN) ≈	NaN (NaN) ≈	NaN (NaN) ≈	NaN (NaN) ≈
RWMOP43	NaN (NaN) ≈	NaN (NaN) ≈	NaN (NaN) ≈	NaN (NaN) ≈	NaN (NaN) ≈	NaN (NaN) ≈	NaN (NaN) ≈	NaN (NaN) ≈
RWMOP44	NaN (NaN) ≈	NaN (NaN) ≈	NaN (NaN) ≈	NaN (NaN) ≈	NaN (NaN) ≈	NaN (NaN) ≈	NaN (NaN) ≈	NaN (NaN) ≈
RWMOP45	NaN (NaN) ≈	NaN (NaN) ≈	NaN (NaN) ≈	NaN (NaN) ≈	NaN (NaN) ≈	NaN (NaN) ≈	NaN (NaN) ≈	NaN (NaN) ≈
RWMOP46	NaN (NaN) ≈	NaN (NaN) ≈	NaN (NaN) ≈	NaN (NaN) ≈	NaN (NaN) ≈	NaN (NaN) ≈	NaN (NaN) ≈	NaN (NaN) ≈
RWMOP47	NaN (NaN) ≈	NaN (NaN) ≈	NaN (NaN) ≈	NaN (NaN) ≈	NaN (NaN) ≈	NaN (NaN) ≈	NaN (NaN) ≈	NaN (NaN) ≈
RWMOP48	NaN (NaN) ≈	NaN (NaN) ≈	NaN (NaN) ≈	NaN (NaN) ≈	NaN (NaN) ≈	NaN (NaN) ≈	NaN (NaN) ≈	NaN (NaN) ≈
RWMOP49	NaN (NaN) −	NaN (NaN) −	NaN (NaN) −	NaN (NaN) −	NaN (NaN) −	NaN (NaN) −	NaN (NaN) −	2.8979e+0 (2.70e+0)
RWMOP50	NaN (NaN) −	NaN (NaN) −	NaN (NaN) −	1.1224e+3 (6.88e+2) ≈	NaN (NaN) −	1.4319e+3 (9.11e+2) ≈	NaN (NaN) −	9.9820e+2 (4.11e+2)
+/−/≈	0/2/13	0/2/13	0/2/13	0/1/14	0/2/13	0/1/14	0/2/13	

**Table 18:** Mean and standard deviation of HV values on power system optimization problems of RWMOPs; ’NAN’ indicates that no feasible solution was found; best result is highlighted in yellow.

Problem	MOEAD	eMOEA	MOPSO	NSGAII	SPEA2	KnEA	GrEA	CMOCPA
RWMOP37	(NaN) ≈	(NaN) ≈	(NaN) ≈	(NaN) ≈	(NaN) ≈	(NaN) ≈	(NaN) ≈	(NaN) ≈
RWMOP38	(NaN) ≈	(NaN) ≈	(NaN) ≈	(NaN) ≈	(NaN) ≈	(NaN) ≈	(NaN) ≈	(NaN) ≈
RWMOP39	(NaN) ≈	(NaN) ≈	(NaN) ≈	(NaN) ≈	(NaN) ≈	(NaN) ≈	(NaN) ≈	(NaN) ≈
RWMOP40	(NaN) ≈	(NaN) ≈	(NaN) ≈	(NaN) ≈	(NaN) ≈	(NaN) ≈	(NaN) ≈	(NaN) ≈
RWMOP41	(NaN) ≈	(NaN) ≈	(NaN) ≈	(NaN) ≈	(NaN) ≈	(NaN) ≈	(NaN) ≈	(NaN) ≈
RWMOP42	(NaN) ≈	(NaN) ≈	(NaN) ≈	(NaN) ≈	(NaN) ≈	(NaN) ≈	(NaN) ≈	(NaN) ≈
RWMOP43	(NaN) ≈	(NaN) ≈	(NaN) ≈	(NaN) ≈	(NaN) ≈	(NaN) ≈	(NaN) ≈	(NaN) ≈
RWMOP44	(NaN) ≈	(NaN) ≈	(NaN) ≈	(NaN) ≈	(NaN) ≈	(NaN) ≈	(NaN) ≈	(NaN) ≈
RWMOP45	(NaN) ≈	(NaN) ≈	(NaN) ≈	(NaN) ≈	(NaN) ≈	(NaN) ≈	(NaN) ≈	(NaN) ≈
RWMOP46	(NaN) ≈	(NaN) ≈	(NaN) ≈	(NaN) ≈	(NaN) ≈	(NaN) ≈	(NaN) ≈	(NaN) ≈
RWMOP47	(NaN) ≈	(NaN) ≈	(NaN) ≈	(NaN) ≈	(NaN) ≈	(NaN) ≈	(NaN) ≈	(NaN) ≈
RWMOP48	(NaN) ≈	(NaN) ≈	(NaN) ≈	(NaN) ≈	(NaN) ≈	(NaN) ≈	(NaN) ≈	(NaN) ≈
RWMOP49	(NaN) −	(NaN) −	(NaN) −	(NaN) −	(NaN) −	(NaN) −	(NaN) −	0.0000e+0 (0.00e+0)
RWMOP50	(NaN) −	(NaN) −	(NaN) −	1.1690e-2 (6.20e-4) ≈	(NaN) −	1.1492e-2 (5.81e-4) ≈	(NaN) ≈	1.1622e-2 (9.08e-4)
+/−/≈	0/2/13	0/2/13	0/2/13	0/1/14	0/2/13	0/1/14	0/2/13	

**Figure A1:**
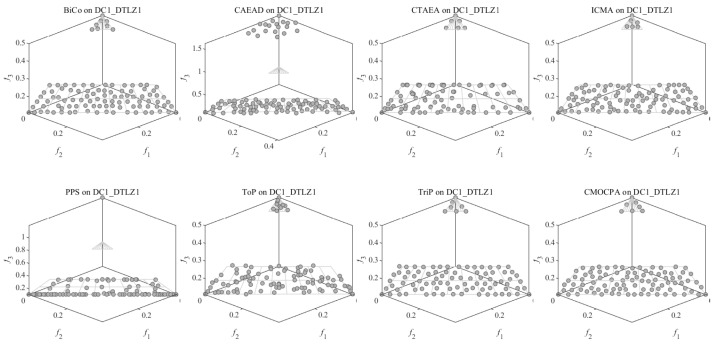
Population with median IGD value among 30 runs obtained by BiCo, CAEAD, C-TAEA, ICMA, PPS, ToP, Trip, and CMOCPA on DC1-DTLZ1.

**Figure A2:**
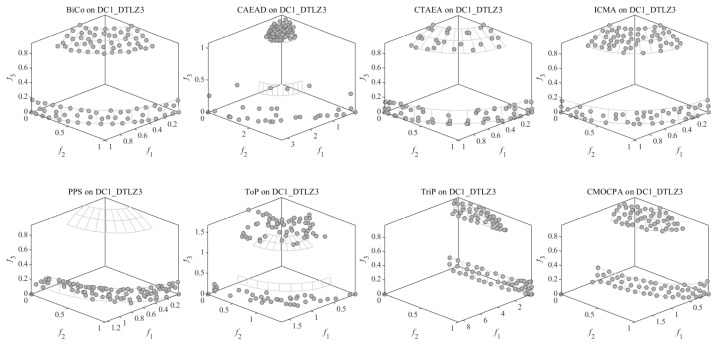
Population with median IGD value among 30 runs obtained by BiCo, CAEAD, C-TAEA, ICMA, PPS, ToP, Trip, and CMOCPA on DC1-DTLZ3.

**Figure A3:**
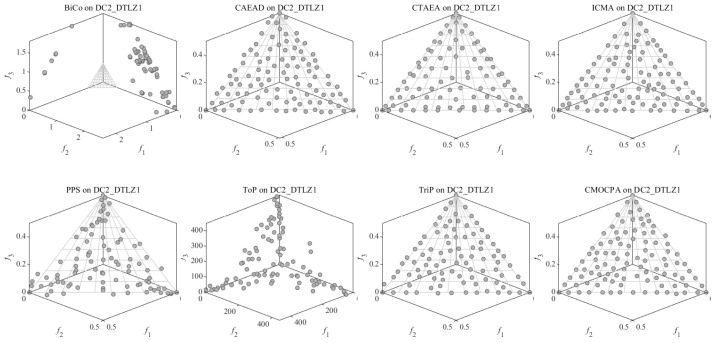
Population with median IGD value among 30 runs obtained by BiCo, CAEAD, C-TAEA, ICMA, PPS, ToP, Trip, and CMOCPA on DC2-DTLZ1.

**Figure A4:**
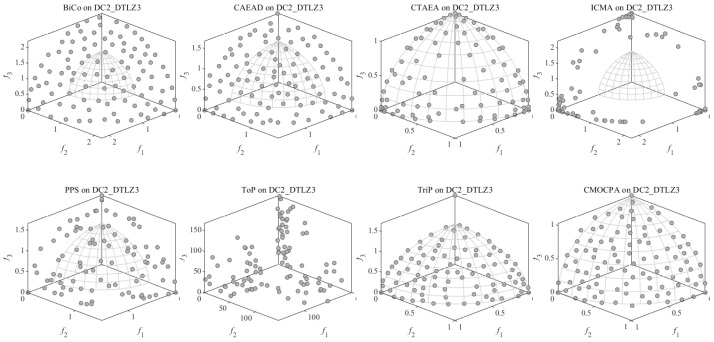
Population with median IGD value among 30 runs obtained by BiCo, CAEAD, C-TAEA, ICMA, PPS, ToP, Trip, and CMOCPA on DC2-DTLZ3.

**Figure A5:**
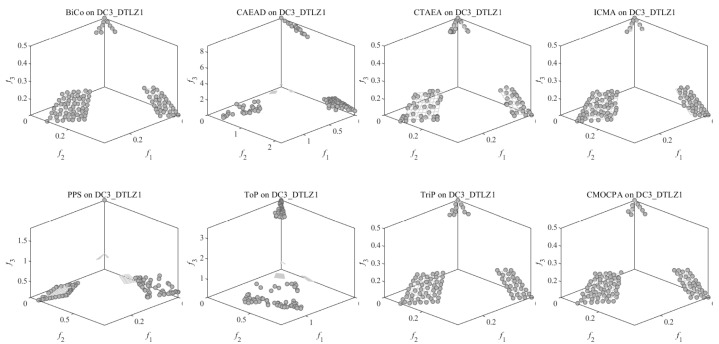
Population with median IGD value among 30 runs obtained by BiCo, CAEAD, C-TAEA, ICMA, PPS, ToP, Trip, and CMOCPA on DC3-DTLZ1.

**Figure A6:**
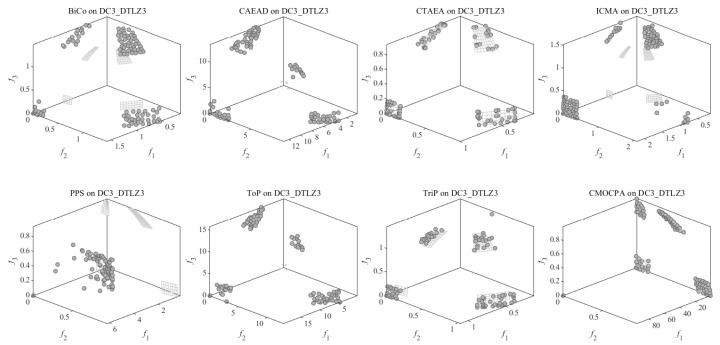
Population with median IGD value among 30 runs obtained by BiCo, CAEAD, C-TAEA, ICMA, PPS, ToP, Trip, and CMOCPA on DC3-DTLZ3.

**Figure A7:**
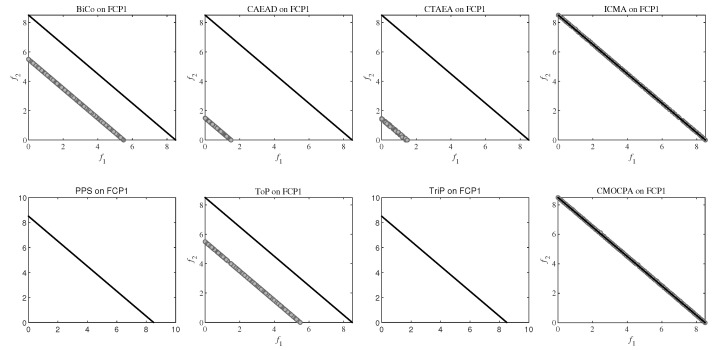
Population with median IGD value among 30 runs obtained by BiCo, CAEAD, C-TAEA, ICMA, PPS, ToP, Trip, and CMOCPA on FCP1.

**Figure A8:**
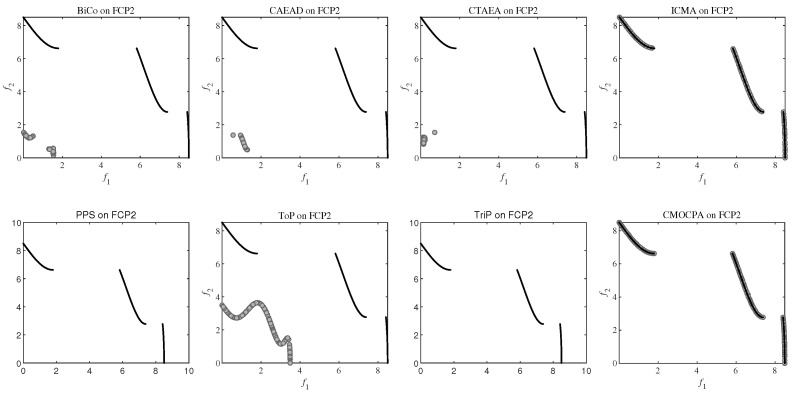
Population with median IGD value among 30 runs obtained by BiCo, CAEAD, C-TAEA, ICMA, PPS, ToP, Trip, and CMOCPA on FCP2.

**Figure A9:**
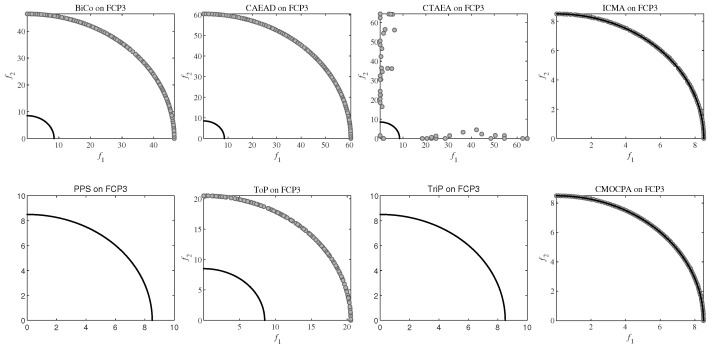
Population with median IGD value among 30 runs obtained by BiCo, CAEAD, C-TAEA, ICMA, PPS, ToP, Trip, and CMOCPA on FCP3.

**Figure A10:**
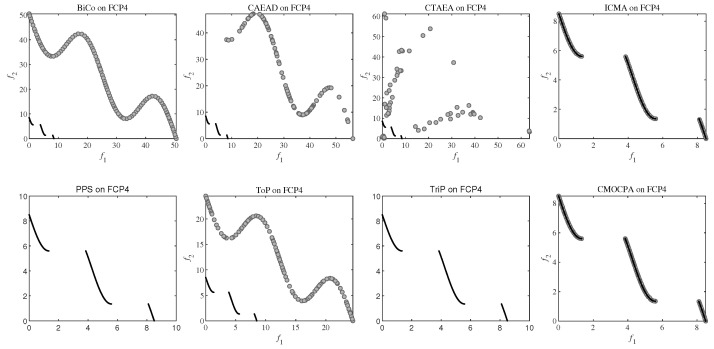
Population with median IGD value among 30 runs obtained by BiCo, CAEAD, C-TAEA, ICMA, PPS, ToP, Trip, and CMOCPA on FCP4.

**Figure A11:**
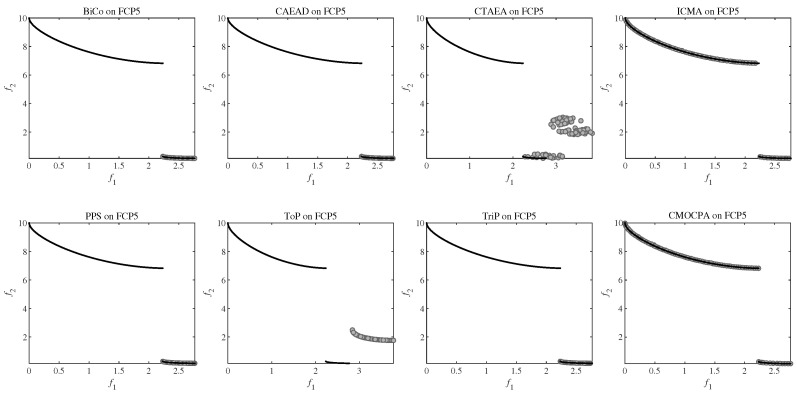
Population with median IGD value among 30 runs obtained by BiCo, CAEAD, C-TAEA, ICMA, PPS, ToP, Trip, and CMOCPA on FCP5.

**Figure A12:**
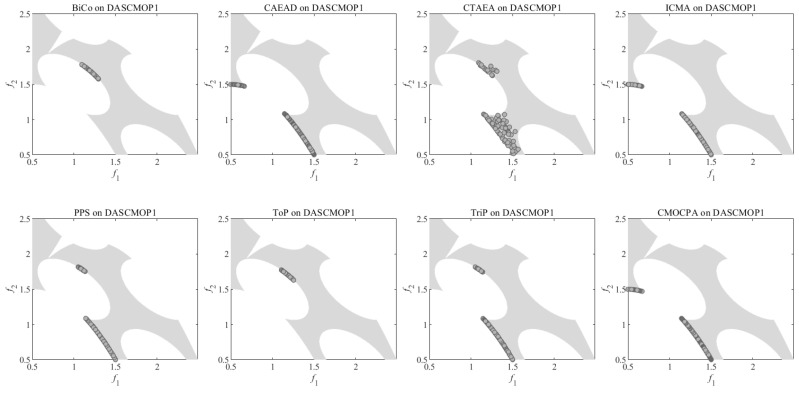
Population with median IGD value among 30 runs obtained by BiCo, CAEAD, C-TAEA, ICMA, PPS, ToP, Trip, and CMOCPA on DASCMOP1.

**Figure A13:**
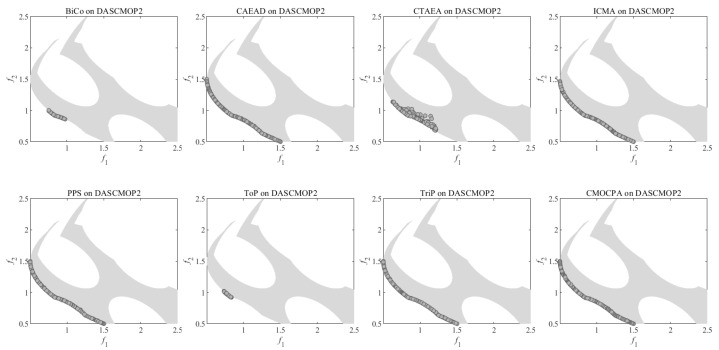
Population with median IGD value among 30 runs obtained by BiCo, CAEAD, C-TAEA, ICMA, PPS, ToP, Trip, and CMOCPA on DASCMOP2.

**Figure A14:**
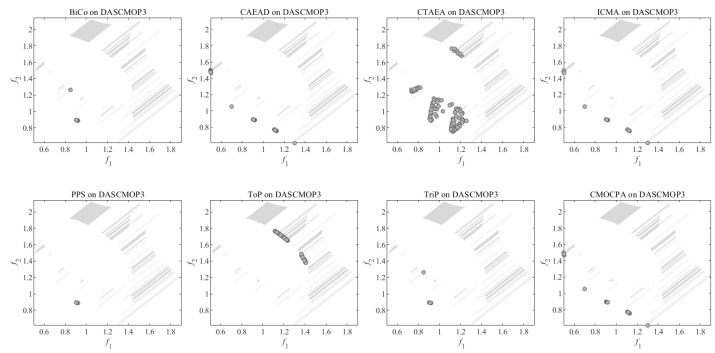
Population with median IGD value among 30 runs obtained by BiCo, CAEAD, C-TAEA, ICMA, PPS, ToP, Trip, and CMOCPA on DASCMOP3.

**Figure A15:**
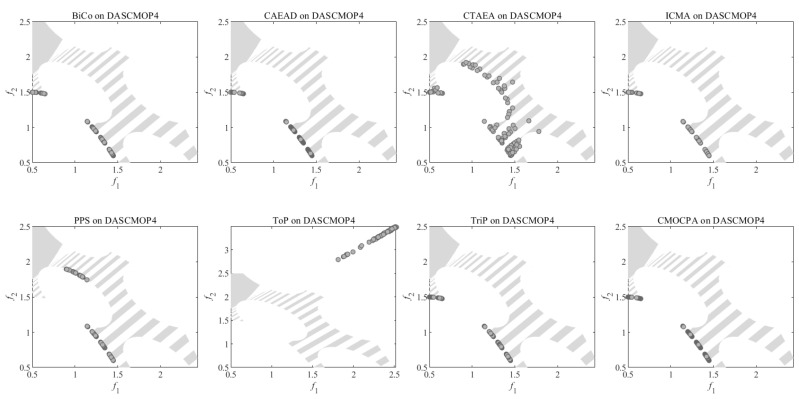
Population with median IGD value among 30 runs obtained by BiCo, CAEAD, C-TAEA, ICMA, PPS, ToP, Trip, and CMOCPA on DASCMOP4.

**Figure A16:**
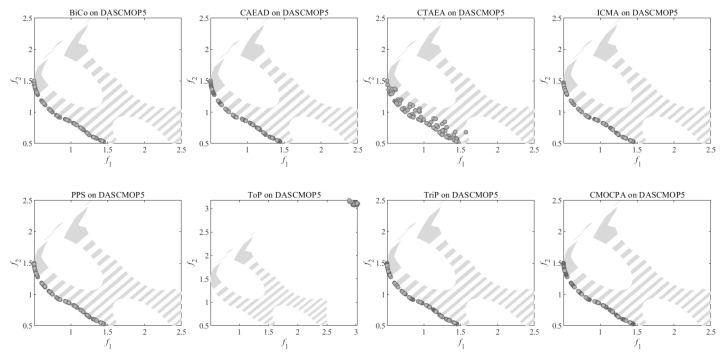
Population with median IGD value among 30 runs obtained by BiCo, CAEAD, C-TAEA, ICMA, PPS, ToP, Trip, and CMOCPA on DASCMOP5.

**Figure A17:**
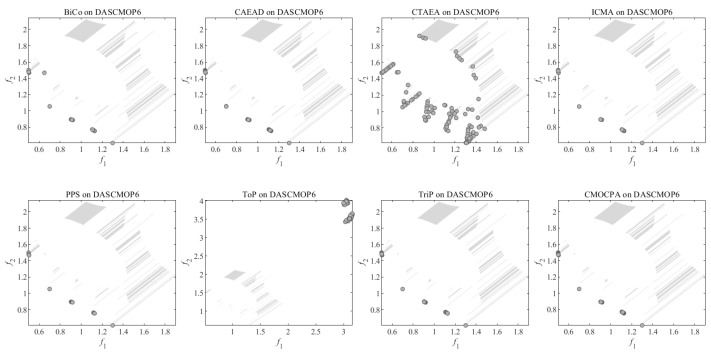
Population with median IGD value among 30 runs obtained by BiCo, CAEAD, C-TAEA, ICMA, PPS, ToP, Trip, and CMOCPA on DASCMOP6.

**Figure A18:**
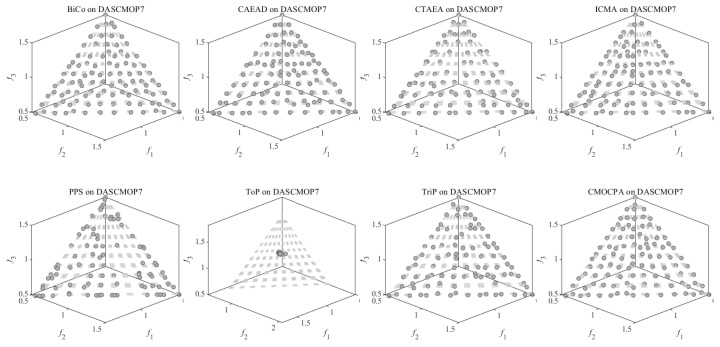
Population with median IGD value among 30 runs obtained by BiCo, CAEAD, C-TAEA, ICMA, PPS, ToP, Trip, and CMOCPA on DASCMOP7.

**Figure A19:**
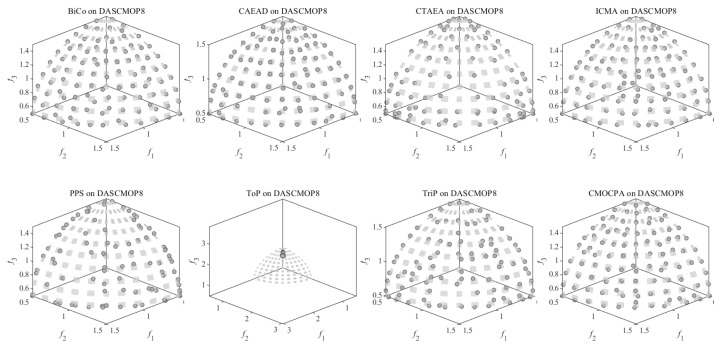
Population with median IGD value among 30 runs obtained by BiCo, CAEAD, C-TAEA, ICMA, PPS, ToP, Trip, and CMOCPA on DASCMOP8.

**Figure A20:**
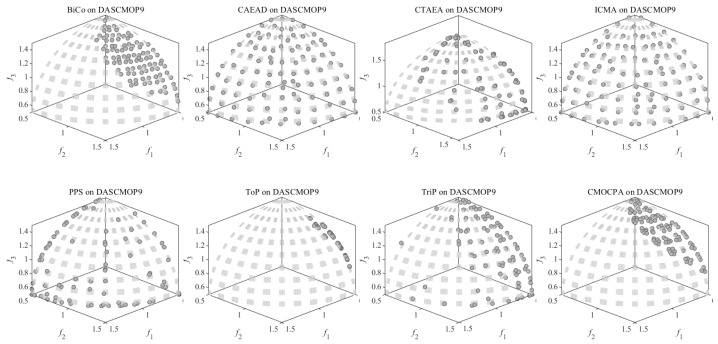
Population with median IGD value among 30 runs obtained by BiCo, CAEAD, C-TAEA, ICMA, PPS, ToP, Trip, and CMOCPA on DASCMOP9.

**Figure A21:**
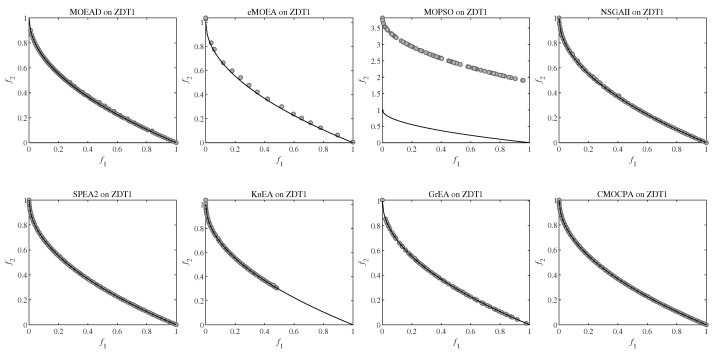
Population with median IGD value among 30 runs obtained by MOEAD, eMOEA, MOPSO, NSGAII, SPEA2, KnEA, GrEA and CMOCPA on ZDT1.

**Figure A22:**
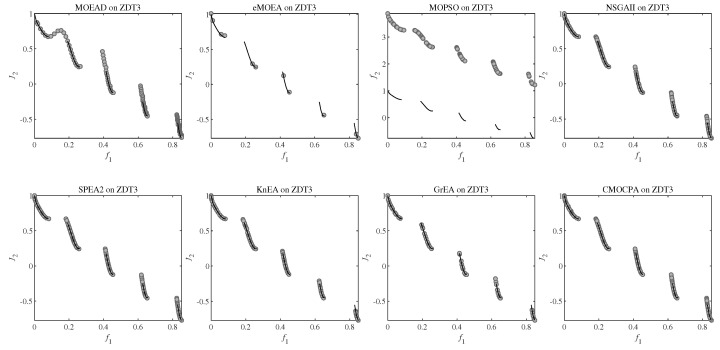
Population with median IGD value among 30 runs obtained by MOEAD, eMOEA, MOPSO, NSGAII, SPEA2, KnEA, GrEA and CMOCPA on ZDT3.

**Figure A23:**
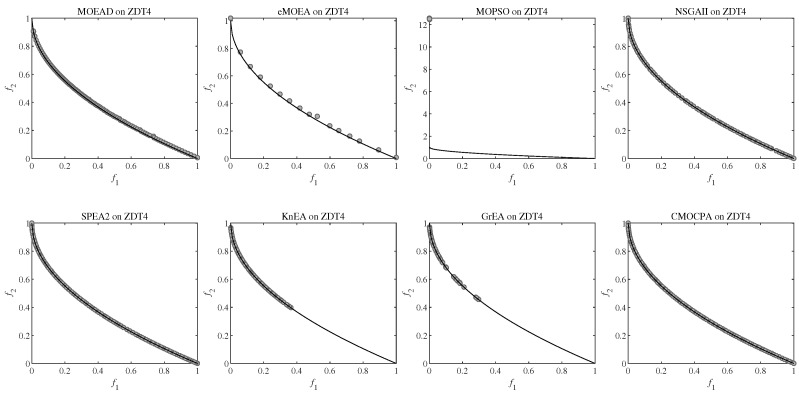
Population with median IGD value among 30 runs obtained by MOEAD, eMOEA, MOPSO, NSGAII, SPEA2, KnEA, GrEA and CMOCPA on ZDT4.

**Figure A24:**
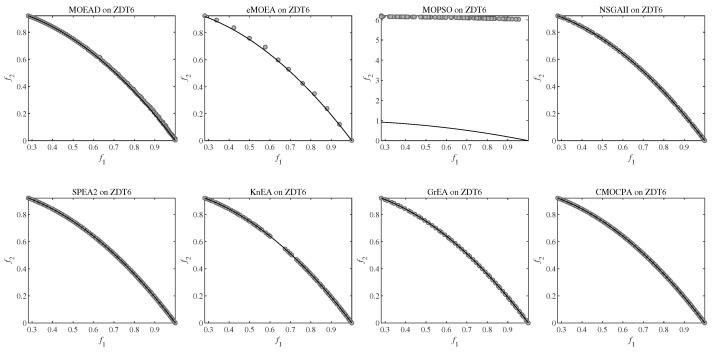
Population with median IGD value among 30 runs obtained by MOEAD, eMOEA, MOPSO, NSGAII, SPEA2, KnEA, GrEA and CMOCPA on ZDT6.

**Figure A25:**
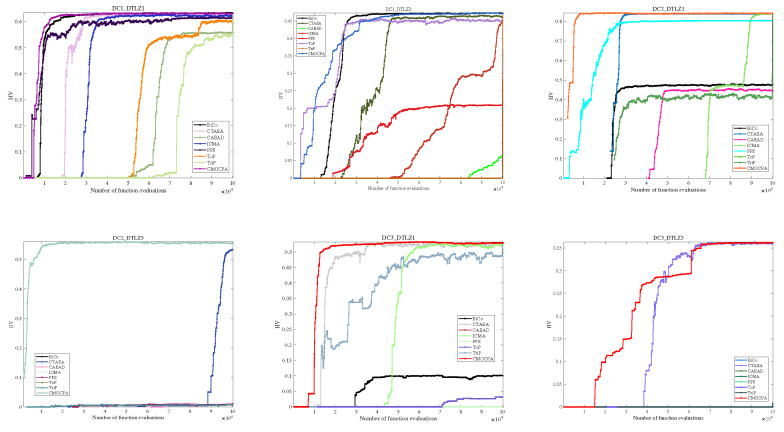
The display of HV indicators obtained by BiCo, CAEAD, C-TAEA, ICMA, PPS, ToP, Trip, and CMOCPA on DC-DTLZ.

**Figure A26:**
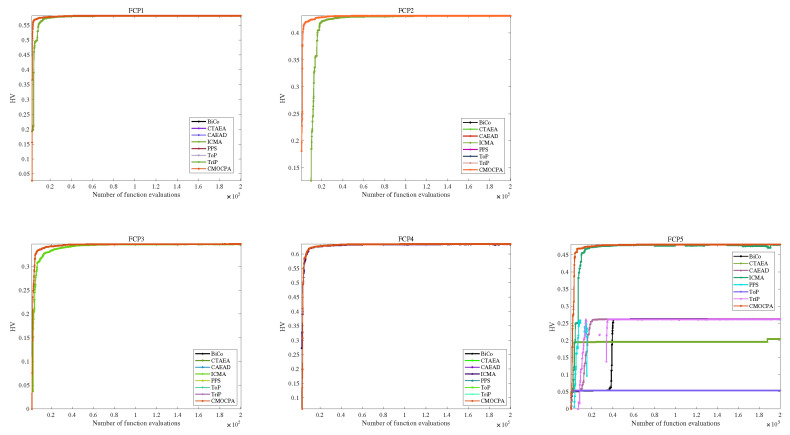
The display of HV indicators obtained by BiCo, CAEAD, C-TAEA, ICMA, PPS, ToP, Trip, and CMOCPA on FCP.

**Figure A27:**
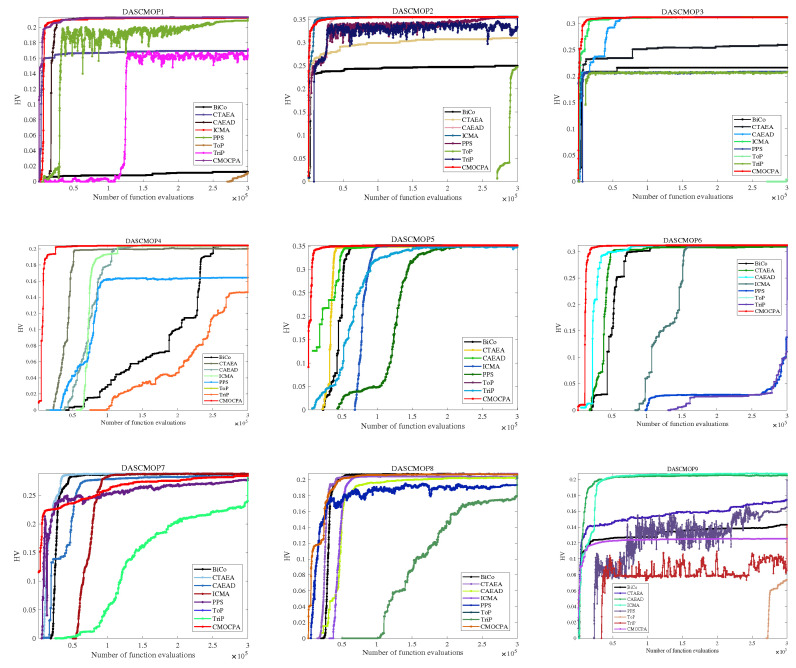
The display of HV indicators obtained by BiCo, CAEAD, C-TAEA, ICMA, PPS, ToP, Trip, and CMOCPA on DASCMOP.

## Data Availability

Data is contained within the article or supplementary material.
